# A new synthetic biology approach allows transfer of an entire metabolic pathway from a medicinal plant to a biomass crop

**DOI:** 10.7554/eLife.13664

**Published:** 2016-06-14

**Authors:** Paulina Fuentes, Fei Zhou, Alexander Erban, Daniel Karcher, Joachim Kopka, Ralph Bock

**Affiliations:** Max-Planck-Institut für Molekulare Pflanzenphysiologie, Potsdam-Golm, Germany; University of British Columbia, Canada

**Keywords:** synthetic biology, metabolic engineering, plastid transformation, combinatorial transformation, Nicotiana tabacum, artemisinin, Other

## Abstract

Artemisinin-based therapies are the only effective treatment for malaria, the most devastating disease in human history. To meet the growing demand for artemisinin and make it accessible to the poorest, an inexpensive and rapidly scalable production platform is urgently needed. Here we have developed a new synthetic biology approach, combinatorial supertransformation of transplastomic recipient lines (COSTREL), and applied it to introduce the complete pathway for artemisinic acid, the precursor of artemisinin, into the high-biomass crop tobacco. We first introduced the core pathway of artemisinic acid biosynthesis into the chloroplast genome. The transplastomic plants were then combinatorially supertransformed with cassettes for all additional enzymes known to affect flux through the artemisinin pathway. By screening large populations of COSTREL lines, we isolated plants that produce more than 120 milligram artemisinic acid per kilogram biomass. Our work provides an efficient strategy for engineering complex biochemical pathways into plants and optimizing the metabolic output.

**DOI:** http://dx.doi.org/10.7554/eLife.13664.001

## eLife digest

Malaria is by far the most devastating tropical disease in the world. It affects hundreds of millions of people – mainly in Africa and Asia – with almost half a million deaths every year. The most effective therapies against malaria all include the drug artemisinin, which is naturally found in an Asian plant called *Artemisia annua*. Unfortunately, the artemisinin content of *A. annua* plants is relatively low and the demand for this drug outstrips the supply of the plant. The costly production process makes artemisinin-based treatments inaccessible to many of the people in the most badly affected regions, and so researchers have been trying to find new ways to produce this drug.

Genetically modifying crop plants, such as tobacco, to produce artemisinin or related compounds could potentially provide a more sustainable and cheaper source of the drug. Inside plant cells, a structure called the nucleus contains DNA that encodes most of a plant’s genes, but compartments called mitochondria and chloroplasts also contain some DNA. Existing methods to genetically modify plants are able to insert a few genes into either the nucleus or the chloroplasts at a time. However, the production of artemisinin in *A. annua* involves many different genes that act at different stages of the process, and the precise roles played by many of them remain unclear.

Fuentes et al. developed a new approach to insert many of the *A. annua* genes involved in artemisinin production into tobacco plants at the same time, instead of one-by-one. The new method, referred to as COSTREL, takes advantage of the researchers’ ability to insert new genes into both the nucleus and the chloroplast of the tobacco plants. In the first step, Fuentes et al. inserted a core set of genes that are essential to make artemisinin into the chloroplast. This enabled the plants to produce a molecule called artemisinic acid, which the researchers can extract from the plants and convert into artemisinin by simple chemical reactions.

After testing different arrangements of the genes in the chloroplast, the plant line that had the highest levels of artemisinic acid was used to introduce a set of “accessory” genes into the nuclear DNA. These accessory genes are not strictly required to make the drug, but they help to regulate the process in a largely unknown manner. The experiments generated hundreds of genetically modified plant lines that each have different combinations of the accessory genes. Fuentes et al. examined these lines and were able to identify plants that could produce large amounts of artemisinic acid. Therefore, these findings lay the foundations for a cheap way to produce this life-saving drug in tobacco. In the future, the COSTREL method developed by Fuentes et al. could also be used to genetically engineer other complex biochemical processes into plants.

**DOI:** http://dx.doi.org/10.7554/eLife.13664.002

## Introduction

**Figure 1.**
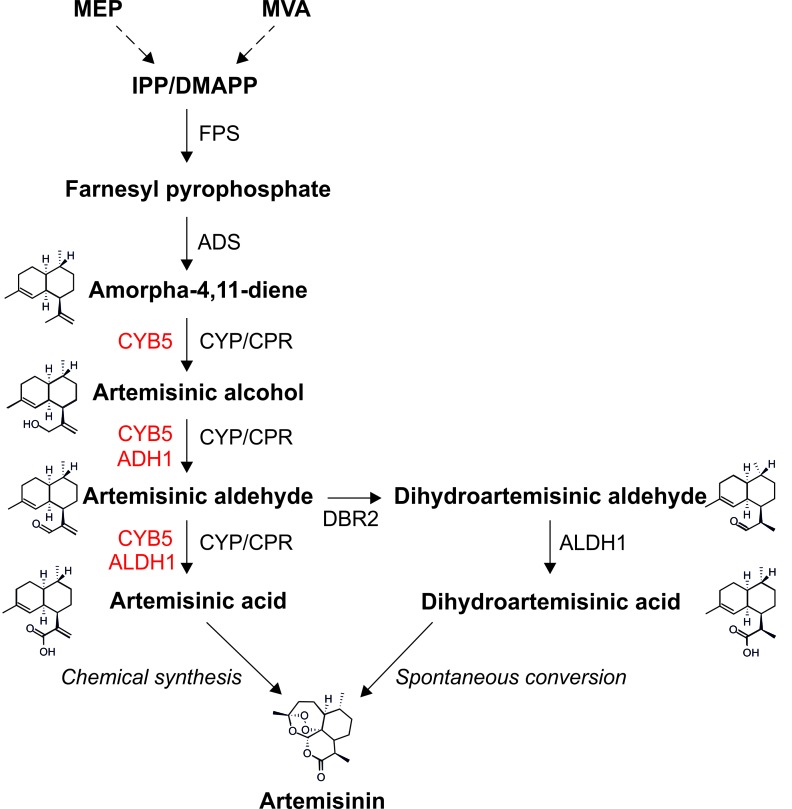
Metabolic pathway of artemisinin biosynthesis. The canonical pathway of artemisinin synthesis starts with the conversion of IPP/DMAPP (C_5_ isoprenoids produced by the MVA pathway in the cytosol or the MEP pathway in the chloroplast) into farnesyl pyrophosphate (FPP), catalyzed by farnesyl pyrophosphate synthase (FPS). Amorpha-4,11-diene synthase (ADS) converts FPP into amorpha-4,11-diene in the first committed step of the pathway. Amorpha-4,11-diene is then successively oxidized to artemisinic alcohol, artemisinic aldehyde and artemisinic acid by the cytochrome P450 monooxygenase CYP71AV1 (CYP) and its redox partner, the cytochrome P450 reductase (CPR). In *A. annua*, artemisinic aldehyde is converted to dihydroartemisinic aldehyde by DBR2, and then to dihydroartemisinic acid by ALDH1. Artemisinin is generated by the spontaneous oxidation of dihydroartemisinic acid *in planta*, and can be produced by chemical conversion of artemisinic acid in vitro. Enzymes depicted in red improve the efficiency of different oxidation steps in yeast (Paddon et al., 2013; Paddon and Keasling, 2014). See text for details. **DOI:** http://dx.doi.org/10.7554/eLife.13664.003

Artemisinin, a C_15_ isoprenoid (sesquiterpene) naturally produced in the wild plant *Artemisia annua* (sweet wormwood, native to temperate Asia), is the main ingredient of artemisinin combination therapies (ACTs), currently the only effective cure of malaria (Okell et al., 2014). As ACTs are the mainstay of malaria treatment and no alternative to artemisinin derivatives is expected to enter the market in the foreseeable future, there is a steadily increasing demand for ACTs which reached nearly 400 million treatment courses in 2013 (http://www.who.int/malaria/publications/world_malaria_report_2014). The mechanism of action of artemisinin on the malaria parasites *Plasmodium falciparium* and *P. vivax* is not entirely clear, but it is generally believed that the reactive endoperoxide bridge present in the molecule (Figure 1) is responsible for its medicinal properties. In addition to their antimalarial activity, artemisinin and its derivatives are currently also considered as promising anti-cancer, antiviral and anti-inflammatory agents (e.g., Willoughby et al., 2009). In *A. annua*, artemisinin is produced in the cytosol of the glandular trichomes of leaves and flowers (Tang et al., 2014). The biosynthesis initiates with the conversion of the isoprenoid building blocks IPP and DMAPP into farnesyl pyrophosphate (FPP) which is then converted into amorpha-4,11-diene by amorphadiene synthase (ADS), the enzyme catalyzing the first committed step of the pathway (Figure 1). Amorpha-4,11-diene is a volatile compound that is oxidized to artemisinic alcohol and subsequently to artemisinic aldehyde by the cytochrome P450 monooxygenase CYP71AV1 (CYP) and its redox partner, the cytochrome P450 reductase (CPR). Artemisinic aldehyde is then further oxidized to artemisinic acid by the same enzyme pair, or alternatively, is reduced to dihydroartemisinic aldehyde by the double bond reductase 2 (DBR2; Figure 1; Zhang et al., 2008). Artemisinic acid can be efficiently and cheaply converted to artemisinin by chemical means (Paddon et al., 2013; Kopetzki et al., 2013) and, therefore, represents a high-value precursor for the industrial production of artemisinin-based pharmaceuticals (Paddon and Keasling, 2014).

In view of the great medicinal value of artemisinic compounds, their low accumulation levels in *A. annua* and the unstable supply of the plant, enormous efforts have been undertaken to produce artemisinic compounds synthetically or in heterologous biological systems. Currently, the semisynthetic synthesis in yeast (Paddon et al., 2013) represents the most efficient heterologous production system for artemisinic acid, the immediate precursor of artemisinin (Figure 1). However, the production costs are still high and ACTs remain unaffordable to many people in the tropical and subtropical regions of Africa and Asia that are most severely afflicted with malaria. Since production in yeast requires large volumes of costly synthetic culture media and large-capacity bioreactors run under sterile conditions, production in plants can potentially provide a much cheaper, renewable and easily scalable source of artemisinic acid. Although the production of artemisinic compounds at low levels has been shown to be feasible in heterologous plant systems (Wu et al., 2006; van Herpen et al., 2010; Zhang et al., 2011; Farhi et al., 2011), the development of an efficient production system for the drug precursor artemisinic acid has not been achieved.

Here we have pursued a novel synthetic biology approach towards high-level production of artemisinic acid in chloroplasts of tobacco (*Nicotiana tabacum*), a fast-growing crop that produces high amounts of biomass at very low cost. We show that by implementing the core pathway in the chloroplast and subsequently selecting for optimum combinations and expression levels of additional pathway enzymes from a large population of combinatorially supertransformed transplastomic lines, artemisinic acid can be produced in tobacco leaves to levels of more than 120 mg/kg fresh weight.

## Results

### Expression of the core pathway for artemisinic acid synthesis from the plastid genome

**Figure 2.**
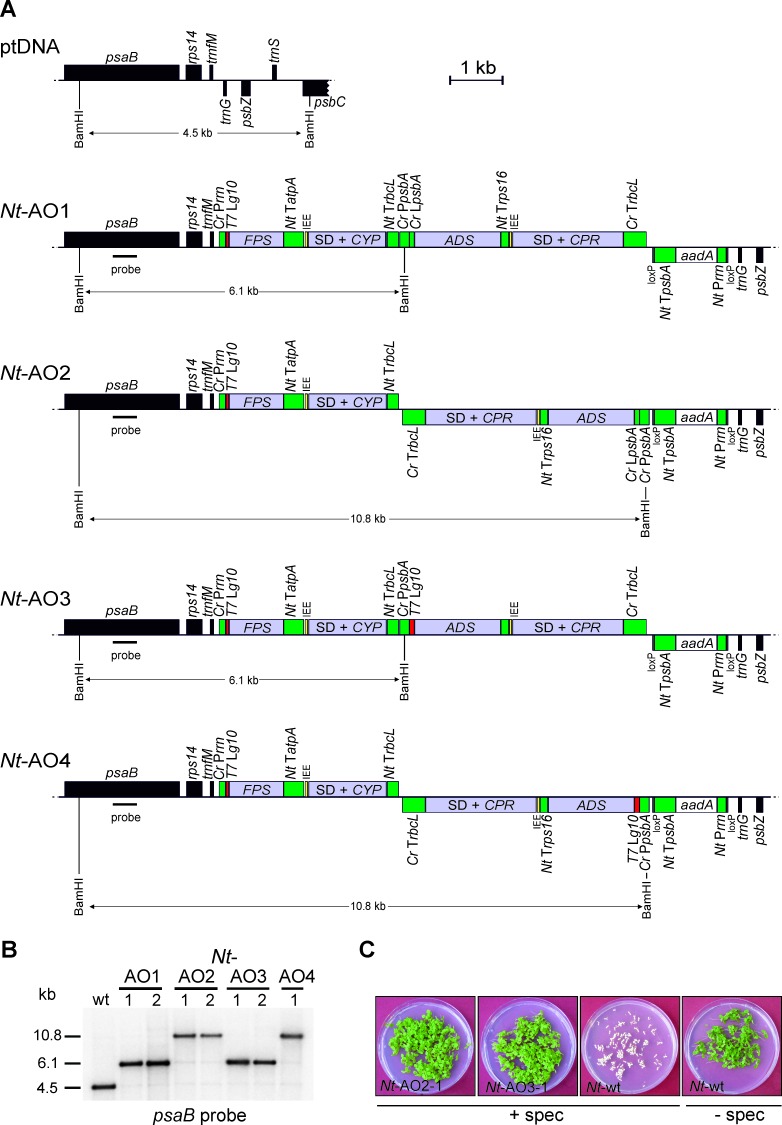
Implementation of the canonical pathway of artemisinic acid biosynthesis in chloroplasts. Synthetic codon-optimized genes for the four enzymes required to produce artemisinic acid (Figure 1) were introduced into the tobacco plastid genome by stable genetic transformation with four different synthetic operon constructs (pAO1-4). The constructs differ in gene arrangement and in the translation signals that drive synthesis of the key pathway enzyme (ADS) catalyzing the first committed step. (**A**) Physical map of the plastid genome region (ptDNA) used for integration of the synthetic artemisinic acid operons and maps of the transgenic loci in the generated transplastomic tobacco lines (*Nt*-AO1-4). The artemisinic acid operon genes are depicted as light blue boxes. Chloroplast promoters and terminators are shown in green, the *aadA* selectable marker gene for chloroplast transformation is represented as a white box, and genes in flanking plastid sequences used for transgene targeting via homologous recombination are in black. Genes above the line are transcribed from left to right, genes below the line are transcribed in the opposite direction. The four transgenes are arranged in two dicistronic operons. *FPS* and *CYP* are driven by the *Chlamydomonas reinhardtii* plastid ribosomal RNA operon promoter (*Cr* P*rrn*) and the *g10* leader sequence from phage T7 (*T7* L*g10*). The second operon containing *ADS* and *CPR* is driven by the *C. reinhardtii psbA* promoter (*Cr* P*psbA*) and either the *T7* L*g10* or the *psbA* leader sequence from *C. reinhardtii (Cr* L*psbA*). This operon is arranged either in sense and downstream of the first operon (AO1, 3) or in antisense, downstream of the *aadA* cassette (AO2, 4). The genes in each operon are separated by an intercistronic expression element (IEE) conferring intercistronic RNA processing and, in this way, enhancing expression of downstream cistrons of the operon (Zhou et al., 2007; Drechsel and Bock, 2010). The BamHI restriction sites used in RFLP analyses and the expected fragment sizes are indicated. The location of the hybridization probe is shown as a black bar. *Cr: C. reinhardtii; Nt: N. tabacum; T7*: bacteriophage T7; P: promoter; L: leader sequence; T: terminator; SD: Shine-Dalgarno sequence. (**B**) RFLP analysis of transplastomic plants. Two independently isolated transplastomic lines are shown for constructs pAO1-3 and one for pAO4. (**C**) Seed assays confirming the homoplasmic state of the transplastomic plants. Seeds were germinated on medium containing 500 mg/L spectinomycin (*Nt*-AO2-1, *Nt*-AO3-1, *Nt*-wt) or antibiotic-free medium (*Nt*-wt). **DOI:** http://dx.doi.org/10.7554/eLife.13664.004

The core enzymes to synthesize artemisinic acid are FPS, ADS, CYP and CPR (Figure 1). Accessory enzymes (indicated in red in Figure 1) and additional enzymes facilitating more efficient biosynthesis of artemisinin are CYB5, ADH1, ALDH1 and DBR2. We first implemented the canonical pathway from FPP to artemisinic acid in tobacco chloroplasts using stable plastid genome transformation (Svab and Maliga, 1993; Bock, 2015). To this end, we designed a number of synthetic operons (Zhou et al., 2007; Lu et al., 2013) that combine the genes for the four core enzymes (FPS, ADS, CYP and CPR; Figure 1) in different arrangements and under the control of different expression signals (Figure 2A). Four synthetic artemisinic acid operon constructs (pAO1-4) were built and introduced into the chloroplast (plastid) genome of tobacco plants by particle gun-mediated transformation. Chloroplast-transformed (transplastomic) lines were selected on regeneration medium with spectinomycin and purified to homoplasmy by additional rounds of selection and regeneration (Svab and Maliga, 1993; Bock, 2015). Restriction fragment length polymorphism (RFLP) analysis verified integration of the synthetic operon constructs into the plastid genome by homologous recombination and successful elimination of all wild-type copies of the highly polyploid chloroplast genome (Figure 2B). Homoplasmy of the transplastomic lines was additionally verified by seed assays that confirmed lack of segregation of the spectinomycin resistance and uniparentally maternal inheritance (Figure 2C).

**Figure 3.**
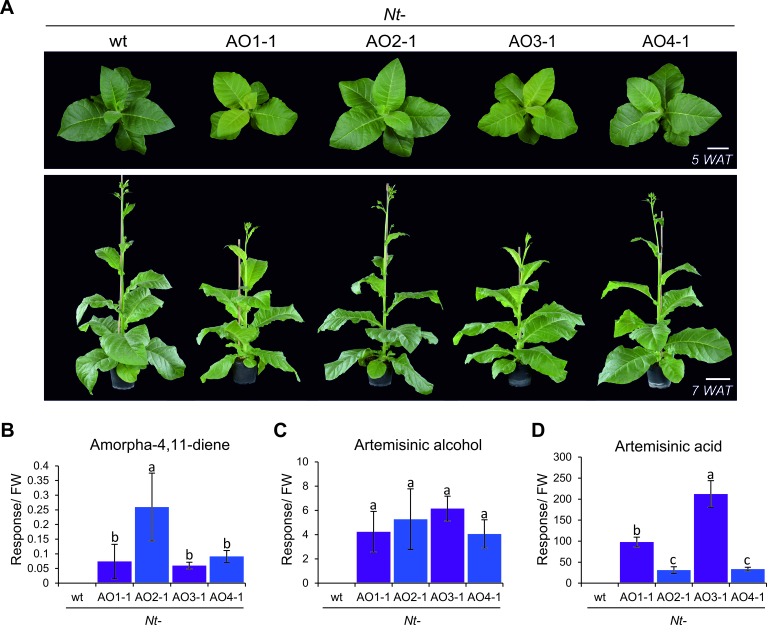
Phenotype of transplastomic tobacco plants and accumulation of artemisinic compounds. (**A**) Transplastomic lines *Nt*-AO1-1 and *Nt*-AO3-1 display a slightly pale and growth-delayed phenotype at the juvenile stage. WAT: weeks after transfer from tissue culture to soil; scale bars: 10 cm. (**B**) Amorpha-4,11-diene is synthesized in all transplastomic lines, but accumulates to lower levels in the lines displaying an altered phenotype (purple bars). (**C**) Artemisinic alcohol is detected in similar amounts in all transplastomic plants. (**D**) Accumulation of artemisinic acid correlates with the altered phenotype of *Nt*-AO1-1 and *Nt*-AO3-1. Relative accumulation of amorpha-4,11-diene was profiled by GC-MS analysis of volatile organic compounds (VOCs). Relative accumulation of the sum of free and conjugated artemisinic alcohol and artemisinic acid were determined by GC-MS analysis of the soluble metabolite fraction after saponification (see Materials and methods; Figures 6 and 7). In agreement with previous reports (van Herpen et al., 2010), these compounds were found to be present mainly as conjugates. Expanding leaves of 5–6 plants per line were used for each measurement. Error bars represent the SD. Different letters above the bars indicate significant differences as determined by One-way ANOVA (p<0.001) and the Holm-Sidak post-hoc test. **DOI:** http://dx.doi.org/10.7554/eLife.13664.005

**Figure 4.**
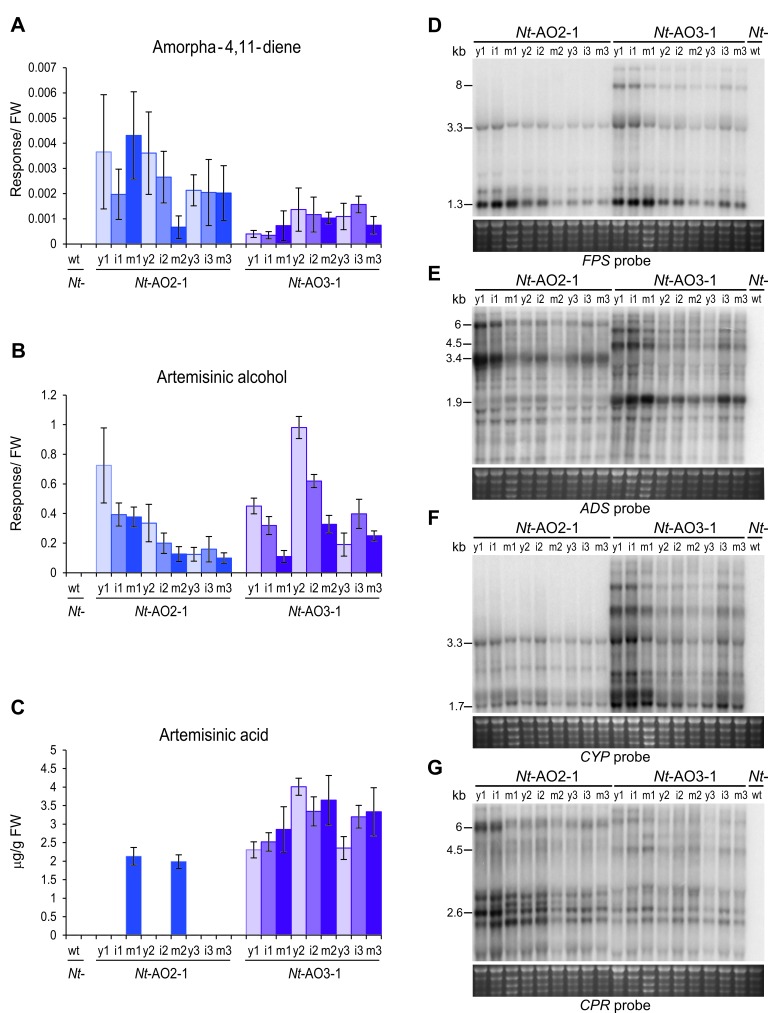
Production of artemisinic acid is maintained throughout plant development and correlates with a high *CYP/CPR* expression ratio. Artemisinic compounds and expression levels of the transgenes were measured in young (stage 1), flowering (stage 2) and old plants (stage 3; see Figure 3—figure supplement 1). (**A**) Amorpha-4,11-diene accumulates to higher levels in line *Nt*-AO2-1 than in *Nt*-AO3-1. (**B**) Artemisinic alcohol is present at similar levels in early (1) and late stages (3) of development in lines *Nt*-AO2-1 and *Nt*-AO3-1, but it is slightly higher in the flowering stage (2) of line *Nt*-AO3-1. (**C**) Artemisinic acid accumulates to high levels during all developmental stages of line *Nt*-AO3-1, whereas in line *Nt*-AO2-1, it is detectable only in mature leaves of young and flowering plants. Relative accumulation of amorpha-4,11-diene and artemisinic alcohol was profiled, the tissue content of artemisinic acid was quantified using an authenticated reference standard (n = 5–6 plants per line; Figures 6 and 7). The sum of free and conjugated artemisinic alcohol and artemisinic acid were determined. y: young leaf; i: expanding (intermediate) leaf; m: fully expanded (mature) leaf. Error bars represent the SD. (**D–G**) Northern blot analysis of the expression of the four transgenes. Total RNA samples from *N. tabacum* wild-type (*Nt*-wt) plants and the transplastomic lines *Nt*-AO2-1 and *Nt*-AO3-1 (at the developmental stages 1–3) were separated in denaturing 1.5% agarose gels, blotted and hybridized to strand-specific RNA probes. Below each blot, the rRNA-containing region of the ethidium bromide-stained gel prior to blotting is shown as a control for RNA integrity and equal loading. The *Nt*-wt sample corresponds to RNA extracted from a fully expanded leaf of a *N. tabacum* wild-type plant at developmental stage 2. The smallest labeled band in each blot corresponds to the monocistronic mRNA. Larger bands represent unprocessed polycistronic precursor transcripts and read-through transcripts (which are common in plastids; e.g., Elghabi et al., 2011; Lu et al., 2013). *CYP* transcripts accumulate to higher levels in line *Nt*-AO3-1, while *CPR* transcripts accumulate to higher levels in line *Nt*-AO2-1, resulting in a higher *CYP/CPR* expression ratio in line *Nt*-AO3-1. **DOI:** http://dx.doi.org/10.7554/eLife.13664.008

All homoplasmic transplastomic lines grew autotrophically under greenhouse conditions and produced viable seeds. However, transplastomic lines obtained with constructs pAO1 and pAO3 (Figure 2A) displayed a slightly pale-green phenotype and a subtle growth delay at the juvenile stage (Figure 3A; Figure 3—figure supplement 1). This phenotype could be due to toxicity of artemisinic metabolites produced in these plants (Bharati et al., 2012) or, alternatively, depletion of isoprenoid precursors from other metabolic pathways in the cell, such as carotenoid and chlorophyll biosyntheses. Measurement of chlorophylls and carotenoids confirmed that, indeed, both pigment classes are significantly reduced in plants exhibiting the mild phenotype (Figure 3—figure supplements 1 and 2). Metabolite profiling (see Materials and Methods) of the transplastomic lines revealed that all lines accumulated the volatile artemisinic acid precursor amorpha-4,11-diene and its first oxidation product artemisinic alcohol (Figure 1; Figure 3). Interestingly, amorpha-4,11-diene accumulated to lower levels in the lines displaying the subtle phenotype, whereas artemisinic alcohol was detected in similar amounts in all transplastomic plants. Accumulation of artemisinic acid correlated with the altered phenotype in *Nt*-AO1-1 and *Nt*-AO3-1, suggesting that a more efficient conversion of amorpha-4,11-diene to downstream metabolites could be the cause of the phenotype. This hypothesis gained support from the analysis of a series of developmental stages and leaf ages which revealed that, while in *Nt*-AO2 plants, artemisinic acid accumulates only in mature leaves of young and flowering plants, it accumulates throughout development in *Nt*-AO3 plants. These analyses also confirmed the inverse relationship between artemisinic acid and amorpha-4,11-diene accumulation (Figure 4A–C).

**Figure 3—figure supplement 1.**
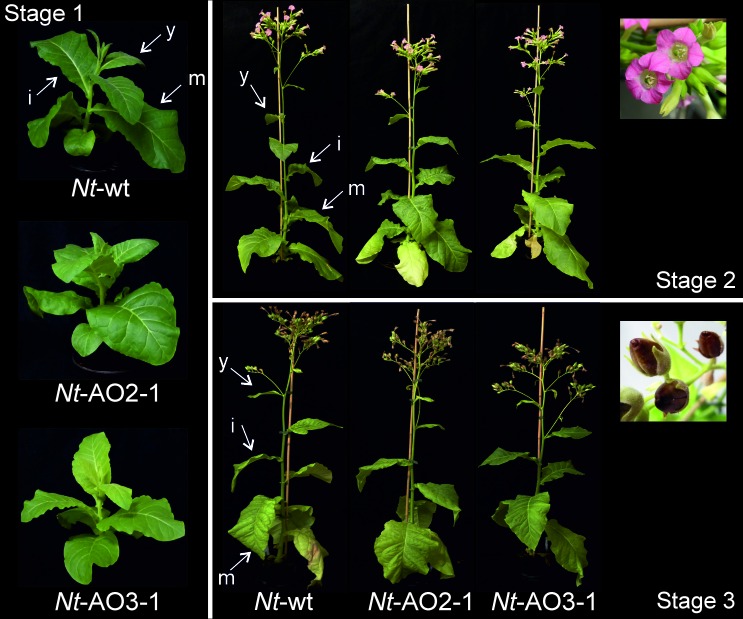
Phenotypes of *Nt*-AO2-1 and *Nt*-AO3-1 plants throughout development. Six plants per line (*Nt*-wt, *Nt*-AO2-1 and *Nt*-AO3-1) were grown under standard greenhouse conditions and photographs were taken of one representative plant per line at different time points: young plants (before flowering, stage 1), flowering plants (stage 2) and old plants (seed capsules formed, stage 3). Light-green leaves and slightly delayed growth of line *Nt*-AO3-1 are more evident at the young stage. At later stages, all *Nt*-AO lines display a wild type-like phenotype and produce viable seeds in indistinguishable amounts. y: young leaf; i: expanding (intermediate) leaf; m: fully expanded (mature) leaf. **DOI:** http://dx.doi.org/10.7554/eLife.13664.006

**Figure 3—figure supplement 2.**
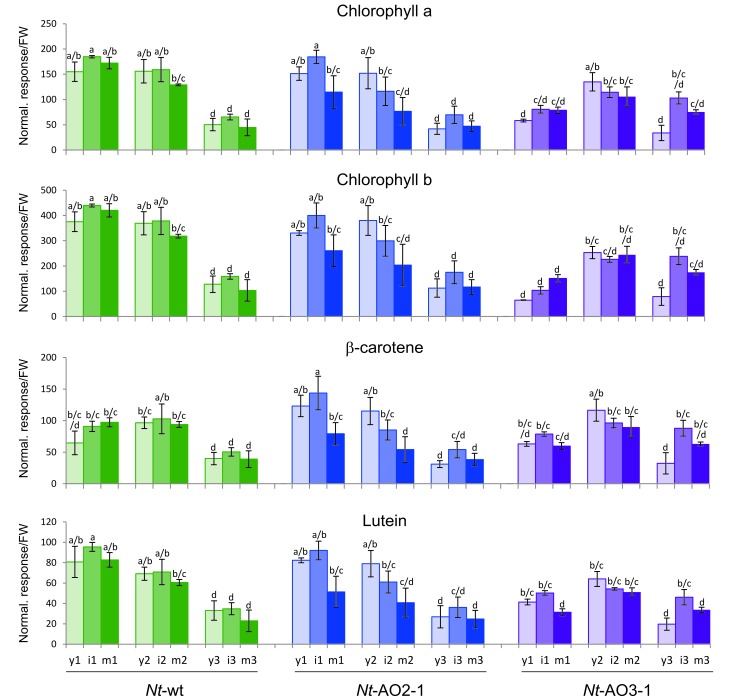
Isoprenoids levels throughout development in wild-type *Nicotiana tabacum* plants (*Nt*-wt) and the transplastomic lines *Nt-*AO2-1 and *Nt-*AO3-1. Plants were grown under standard greenhouse conditions and samples were taken from young (y), expanding (i), and fully expanded (m) leaves at three developmental stages (1–3; cf. Figure 3—figure supplement 1). Metabolite levels were determined by UPLC analysis. The values represent the peak height for each compound divided by 10.000 and normalized to the fresh weight (FW), resulting in the normalized response/FW. Error bars represent the SD (n = 3 plants per line). Different letters above the bars indicate significant differences as determined by One-way ANOVA (p<0.05) and the Holm-Sidak post-test. **DOI:** http://dx.doi.org/10.7554/eLife.13664.007

To identify the molecular basis of the striking metabolic differences between the different operon constructs, a series of northern blot experiments was conducted. In view of the commonalities of the *Nt*-AO2 and *Nt*-AO4 plants versus the *Nt*-AO1 and *Nt*-AO3 plants, it seemed reasonable to assume that the relative orientation of the two operons (Figure 2A) is causally responsible for the different visual and metabolic phenotypes. When the expression of the four transgenes was assayed, a striking difference was observed in the *CYP/CPR* expression ratio in that high artemisinic acid accumulation correlated with a high *CYP/CPR* expression ratio in *Nt*-AO3 plants (Figure 4D–G; Figure 4—figure supplement 1). In nature, CYPs are often found in excess to their CPR counterparts, with ratios of 10–100:1 or higher (reviewed, e.g., in Guengerich, 2002). Even though there is currently no consensus explanation for this observation, it is known that CPRs can activate molecular oxygen, thereby producing superoxide radicals and wasting redox capacity of the cell (Manoj et al., 2010). A high CYP/CPR ratio would prevent this CPR-mediated toxicity and result in a more efficient use of the redox power of the cell for artemisinic acid synthesis, as observed in our *Nt*-AO1 and *Nt*-AO3 transplastomic lines. This explanation is also in agreement with published data on transcript accumulation and protein abundance for these two enzymes in *A. annua*. While the levels of the *CPR* transcript and the CPR protein remain constant during development of the plant and in different organs, the transcript and protein levels of CYP increase in the developmental stages and organs where artemisinin synthesis is induced (Olofsson et al., 2011; Zeng et al., 2008). Especially the final oxidation step from artemisinic aldehyde to artemisinic acid appears to require an effective monooxygenase (Ting et al., 2013), suggesting that the higher levels of artemisinic acid in our transplastomic *Nt*-AO1 and *Nt*-AO3 lines are most likely related to their higher *CYP/CPR* expression ratio. However, determination of the CYP and CPR protein accumulation levels (and enzyme activities) would be necessary to precisely assess these ratios and ultimately confirm their impact on metabolite conversion in the pathway.

**Figure 4—figure supplement 1.**
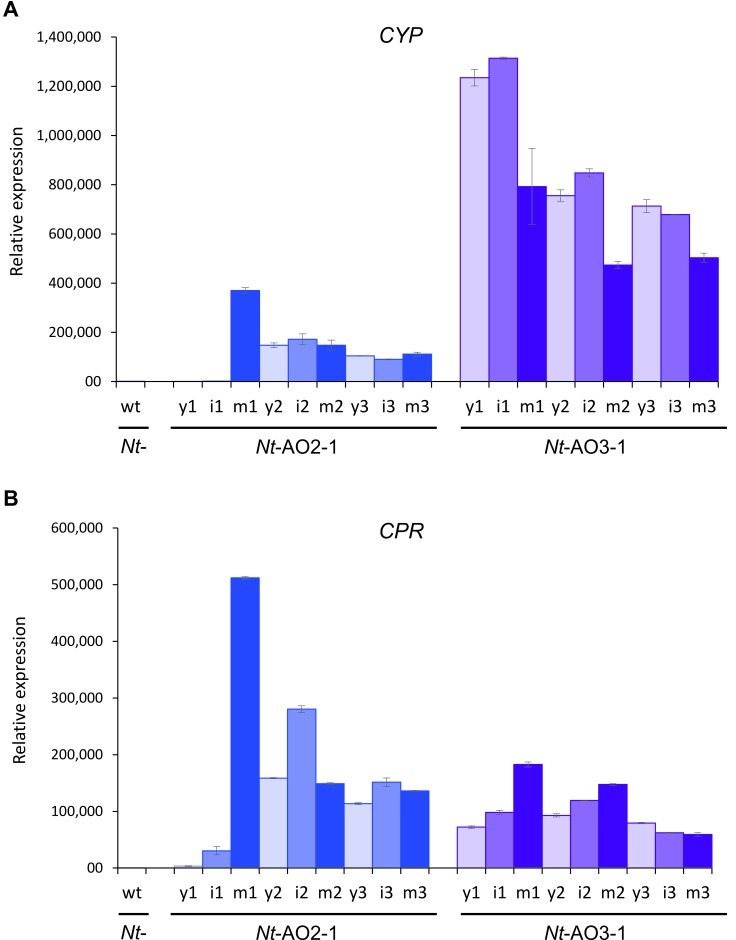
Quantitation of the expression of *CYP* and *CPR* by qRT-PCR analysis. Samples from young (y), expanding (intermediate; i) and fully expanded (mature; m) leaves were measured in three technical replicates for each line, in early, flowering and late developmental stages (1–3; cf. Figure 3—figure supplement 1). The wild-type sample (wt) corresponds to a sample from a fully expanded leaf of *N. tabacum* cv. Petit Havana at the flowering stage. The qRT-PCR data confirm the northern blot analyses that had revealed a higher *CYP* to *CPR* expression ratio in transplastomic line *Nt*-AO3-1 than in line *Nt*-AO2-1. **DOI:** http://dx.doi.org/10.7554/eLife.13664.009

### Pathway optimization by combinatorial supertransformation

Having successfully implemented the canonical pathway of artemisinic acid synthesis into the chloroplast of tobacco plants, we next sought to maximize artemisinic acid production. In our best-performing transplastomic plants (*Nt*-AO3), artemisinic acid accumulation reached a maximum of 2–4 mg/kg fresh weight (FW), equivalent to approximately 20–40 mg/kg dry weight (DW; or 0.002–0.004% DW), a level significantly lower than artemisinin accumulation in *A. annua* (varying between 0.01 and 1% DW; Liu et al., 2011; Bryant et al., 2015). Recently, a number of accessory *A. annua* enzymes have been identified that enhance the flux through the pathway, including a cytochrome b_5_ (CYB5) that promotes electron transfer to P450 monooxygenases (Schenkman and Jansson, 2003), a new alcohol dehydrogenase (ADH1) that improves the oxidation of artemisinic alcohol to artemisinic aldehyde (Paddon et al., 2013) and an aldehyde dehydrogenase (ALDH1) that catalyzes the conversion of dihydroartemisinic aldehyde into dihydroartemisinic acid and, in yeast, also enhances the conversion of artemisinic aldehyde into artemisinic acid (Paddon et al., 2013; Figure 1). We also considered two additional enzymes: The double bond reductase 2 (DBR2) from *A. annua* introduces a branch point into the pathway by reducing artemisinic aldehyde to dihydroartemisinic aldehyde (Zhang et al., 2008) and, therefore, potentially can lead to the synthesis of artemisinin (Figure 1). Finally, the 1-deoxy-D-xylulose-5-phosphate reductoisomerase (DXR) from the cyanobacterium *Synechocystis*, a key regulatory enzyme in the MEP pathway of isoprenoid biosynthesis, was selected because its expression may improve precursor availability (Figure 1). Since the quantitative contributions of these enzyme activities to artemisinic acid biosynthesis are not well understood and, moreover, the optimum enzyme activities required to mediate maximum flux through the pathway are unknown, we decided to pursue a combinatorial supertransformation approach. Combinatorial transformation involves the mixing of multiple single-gene transformation constructs and their biolistic co-transformation followed by large-scale screening of many transgenic lines by their metabolic (or other) phenotypes (Zhu et al., 2008; Naqvi et al., 2009). Individual transgenic lines generated by this approach differ in the transgene combination they harbor as well as in transgene copy numbers and expression levels, thus facilitating selection of optimized genotypes that condition the desired metabolic output (Naqvi et al., 2009). We applied combinatorial nuclear transformation to our transplastomic *Nt*-AO2-1 (high accumulation of amorpha-4,11-diene but low levels of artemisinic acid) and *Nt*-AO3-1 (low accumulation of amorpha-4,11-diene and high accumulation of artemisinic acid) lines, assuming that artemisinic acid production can be substantially increased by identifying the optimum combination and expression levels of the additional pathway enzymes. Combinatorial supertransformation of transplastomic lines encoding a canonical metabolic pathway with a plasmid cocktail containing additional and/or accessory pathway enzymes represents a new approach in synthetic biology that we refer to as COSTREL (for COmbinatorial Supertransformation of Transplastomic REcipient Lines).

Genes for the five candidate enzymes (CYB5, ADH1, ALDH1, DBR2, DXR) were cloned into individual expression cassettes, the resulting plasmids were mixed and co-bombarded with a kanamycin resistance gene into the nuclear genomes of transplastomic *Nt*-AO2-1 and *Nt*-AO3-1 plants. 612 kanamycin-resistant shoots (*Nt-*AO-CS lines) were generated by supertransformation of the transplastomic recipient lines *Nt-*AO2-1 and *Nt-*AO3-1. After rooting in kanamycin-containing medium, 512 plantlets were transferred to soil and grown to maturity under standard greenhouse conditions. At the onset of flowering, a fully expanded leaf was harvested for preliminary profiling of artemisinic acid and its precursors by GC-MS (see Materials and Methods). Based on growth, phenotype and fertility of the plants, 199 COSTREL lines were selected for metabolic screening of artemisinic compounds: 79 *Nt*-AO2-CS and 120 *Nt*-AO3-CS lines (Figure 5—source data 1). The various lines displayed great variation with respect to the accumulation levels of the compounds assayed (amorpha-4,11-diene, artemisinic alcohol, dihydroartemisinic alcohol, dihydroartemisinic acid and artemisinic acid). Importantly, COSTREL lines could be identified that contained strongly increased levels of the drug precursor artemisinic acid (Figure 5—source data 1).

**Figure 5.**
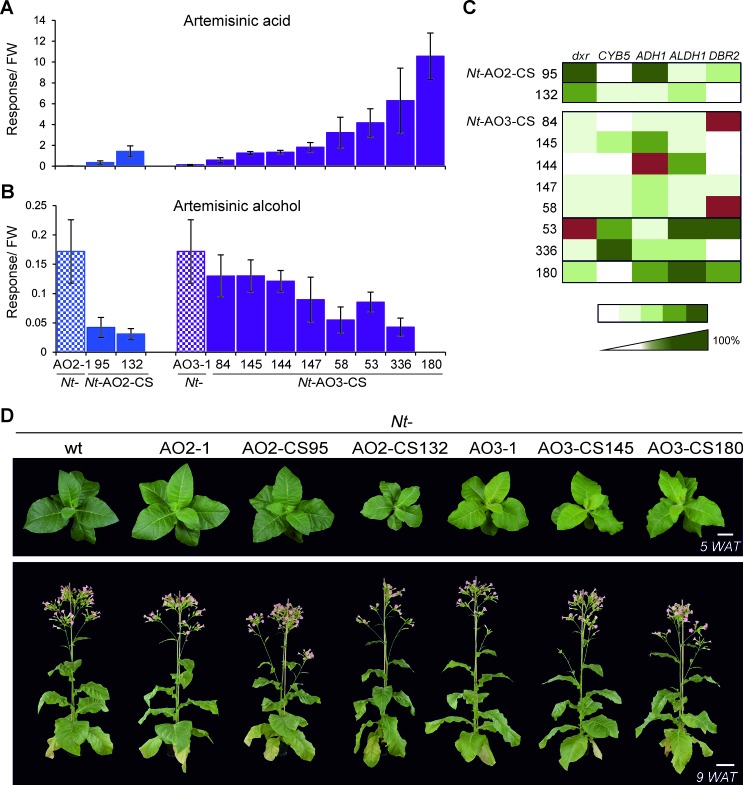
Isolation of combinatorially supertransformed transplastomic lines with a strong increase in artemisinic acid accumulation. Transplastomic lines *Nt*-AO2-1 and *Nt*-AO3-1 were combinatorially supertransformed with genes for additional enzymes of the pathway (Figure 1) to facilitate large-scale screening for increased artemisinic acid production. (**A**) Relative artemisinic acid levels (given in response/FW; see Materials and methods) in the T1 generation of two combinatorially supertransformed lines obtained with transplastomic recipient line *Nt*-AO2-1 (*Nt*-AO2-CS) and eight lines obtained with transplastomic recipient line *Nt*-AO3-1 (*Nt*-AO3-CS). An up to 77-fold increase in artemisinic acid was achieved (line *Nt*-AO3-CS180) in comparison to recipient line *Nt*-AO3-1. For a complete list of screened supertransformed lines, see Figure 5—source data 1. (**B**) Inverse relationship between artemisinic acid accumulation and artemisinic alcohol accumulation in supertransformed lines. Fully expanded leaves of 5–6 plants per line (at the flowering stage) were used for metabolite profiling. The sum of free and conjugated artemisinic alcohol and artemisinic acid were determined. (**C**) qRT-PCR analysis of transgene expression suggests a predominant role of *ALDH1* in boosting artemisinic acid synthesis. 2–3 plants per line were measured, and the expression levels were ranked after One-way ANOVA comparison (p<0.05). Brown color indicates absence of gene expression. (**D**) Combinatorially supertransformed lines with a high increase in artemisinic acid (*Nt*-AO2-CS132 and *Nt*-AO3-CS180) display a similar phenotype as the corresponding transplastomic recipient line. WAT: weeks after transfer from tissue culture to soil; scale bars: 10 cm. **DOI:** http://dx.doi.org/10.7554/eLife.13664.010 10.7554/eLife.13664.011Figure 5—source data 1.Metabolic screening (phenotyping) and genotyping of the T0 generation of combinatorially supertransformed *Nt*-AO-CS lines.The supertransformed lines *Nt*-AO2-CS and *Nt-*AO3-CS are arranged according to their artemisinic acid content, from low to high. Fresh weight corrected response values (R/FW) for amorpha-4,11-diene were multiplied by 1,000 and expressed as Rx1000/FW. Dihydroartemisinic acid was only detectable in line *Nt*-AO3-CS180 at a low level of 0.03 R/FW. Asterisks mark the selected candidate lines further analyzed in the T1 generation. Lines were clustered according to artemisinic acid content using hierarchical cluster analysis based on Ward’s method. Cnd: cluster nd (artemisinic acid not detected); C1: cluster 1; C2: cluster 2; C3: cluster 3; C4: cluster 4; C5: cluster 5. Genes detected in the genomic PCR assays are numbered as follows: 1: *dxr*; 2: *CYB5*; 3: *ADH1*; 4: *ALDH1*; 5: *DBR2*; 0: no gene detected; ?: unclear result. nd: not detected; nm: not measured; -: not determined. Amorpha-4,11-diene values are from one measurement per line, values for artemisinic alcohol, dihydroartemisinic alcohol and artemisinic acid represent averages of three technical replicates per line. SD: standard deviation.**DOI:** http://dx.doi.org/10.7554/eLife.13664.011

In combinatorial transformation, all transgenes that simultaneously enter the nucleus of the recipient cell usually integrate into the same genomic locus (most likely into a transient DNA double-strand break), and therefore co-segregate into the next generation (Naqvi et al., 2009). This feature allowed us to raise a T1 generation of supertransformed lines from seeds and repeat the metabolite profiling with T1 leaf material grown under highly standardized conditions. These analyses confirmed the results obtained with the T0 plants and revealed that, in the case of the *Nt-*AO2-CS lines, the highest increase in artemisinic acid content occurred in line 132 showing a 33-fold increase compared to its transplastomic recipient line *Nt*-AO2-1, whereas among the *Nt*-AO3-CS lines, line 180 reached an even 77-fold increase compared to transplastomic line *Nt*-AO3-1 (Figure 5—source data 1; Figure 5A). The trait artemisinic acid content was stable across generations, and the highest producer, line *Nt-*AO3-CS180, reached levels of 120.4 ± 42 mg per kg FW in the T1 generation.

**Figure 5—figure supplement 1.**
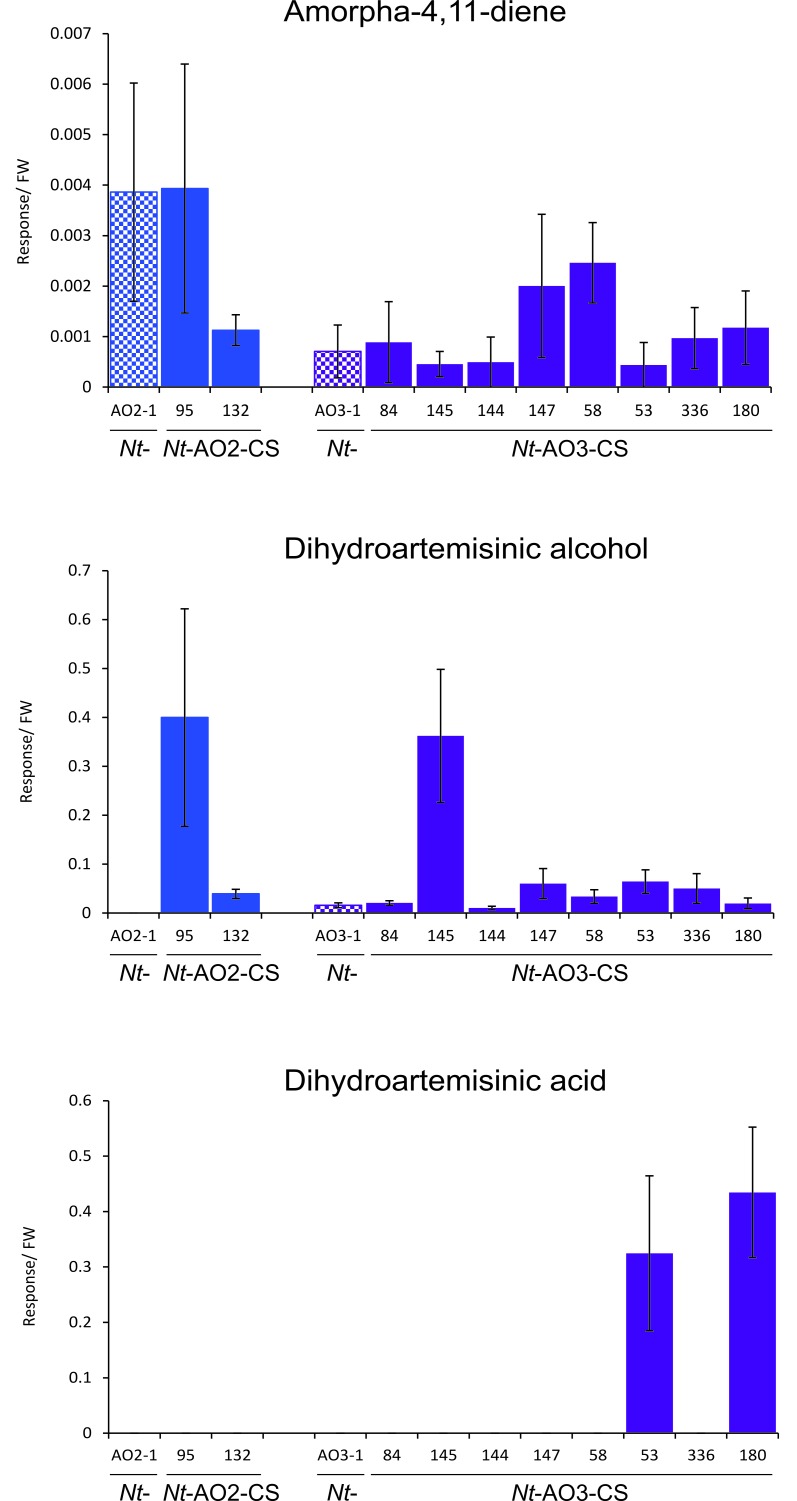
Amorpha-4,11-diene, dihydroartemisinic alcohol and dihydroartemisinic acid accumulation in the T1 generation of combinatorially supertransformed plants. Levels of amorpha-4,11-diene in *Nt*-AO-CS lines do not correlate with the strong increase in artemisinic acid (Figure 5). Dihydroartemisinic alcohol accumulates to higher levels in lines *Nt*-AO2-CS95 and *Nt*-AO3-CS145. Dihydroartemisinic acid is detectable only in two of the best-performing COSTREL lines. Amorpha-4,11-diene levels were determined by GC-MS of VOCs. Dihydroartemisinic alcohol and dihydroartemisinic acid were measured by GC-MS analysis of soluble saponified metabolites. The sum of free and conjugated artemisinic compounds was determined. Error bars represent the SD (n = 5–6 plants per line). **DOI:** http://dx.doi.org/10.7554/eLife.13664.012

**Figure 5—figure supplement 2.**
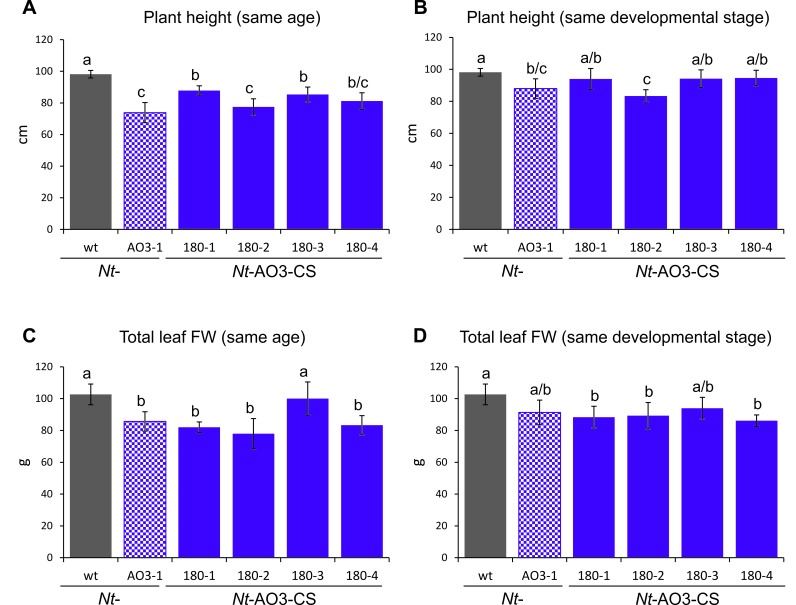
Measurements of plant height and total leaf biomass of COSTREL line *Nt*-AO3-CS180 (progeny of four different T1 lines), its transplastomic progenitor line *Nt*-AO3-1 and the wild type (wt). (**A**) Comparison of plant height at the same age. Plants were measured when the wild type started to flower. (**B**) Comparison of plant height at the same developmental stage. Transplastomic line *Nt*-AO3-1 and COSTREL line *Nt*-AO3-CS180 were measured five days later than the wild type to compensate for their slightly delayed onset of flowering. (**C**) Comparison of total leaf biomass (fresh weight, FW) at the same plant age. Plants were measured when the wild type started to flower. (**D**) Comparison of total leaf biomass at the same developmental stage. Transplastomic line *Nt*-AO3-1 and COSTREL line *Nt*-AO3-CS180 were measured five days later than the wild type to compensate for their delayed flowering. Note that, at the same plant age, transplastomic line *Nt*-AO3-1 and COSTREL line *Nt*-AO3-CS180 are slightly shorter and produce less total leaf biomass than the wild type. Once the transplastomic plants and the COSTREL lines flower (same developmental stage, five days later), the COSTREL plants reach a height and a total leaf biomass that is close to the values measured for the wild type. On average, the COSTREL plants are 7% shorter and produce 13% less leaf biomass than wild-type plants. No significant difference in either height or total leaf FW was observed between COSTREL plants and their transplastomic progenitor line *Nt*-AO3-1. Error bars represent the SD (n = 6). Different letters above the bars indicate significant differences as determined by One-way ANOVA (p<0.05) and the Holm-Sidak post-hoc test. **DOI:** http://dx.doi.org/10.7554/eLife.13664.013

### Identification of limiting steps in artemisinic acid biosynthesis

To obtain insights into pathway regulation and identify bottlenecks in artemisinic acid synthesis, we investigated correlations between pathway metabolites and between artemisinic acid accumulation and the set of transgenes expressed in the nucleus of *Nt*-AO2-CS and *Nt*-AO3-CS COSTREL lines.

**Figure 6.**
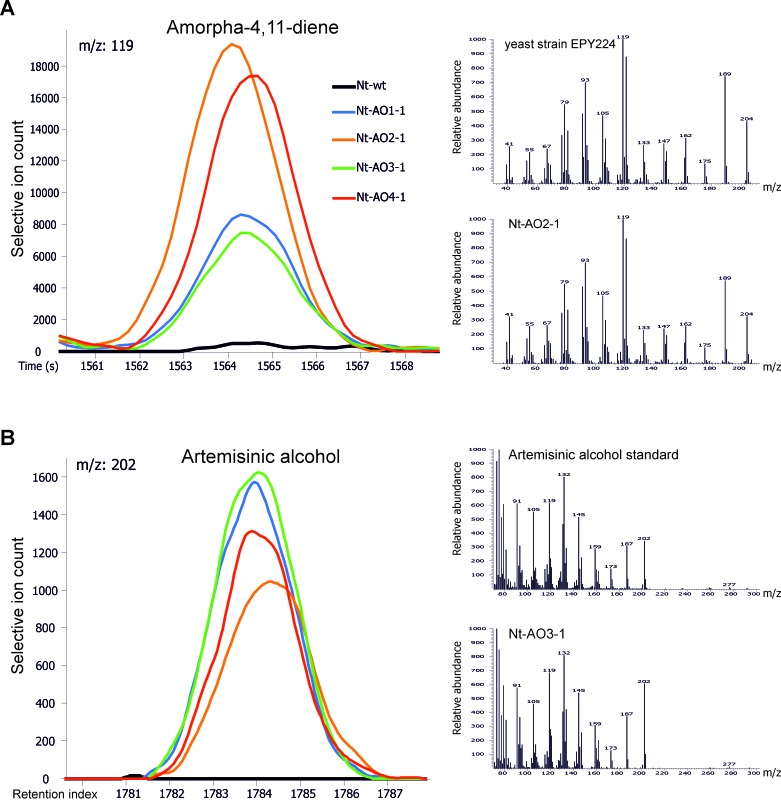
Chromatograms and mass spectra of amorpha-4,11-diene and artemisinic alcohol. Characteristic peaks for one specific fragment at the expected retention time or index are displayed for each compound. (**A**) Amorpha-4,11-diene-specific mass feature 119 at a retention time of 1564 s. This metabolite is present in all *Nt*-AO lines, and at slightly higher levels in lines *Nt*-AO2-1 and *Nt*-AO4-1. (**B**) Artemisinic alcohol-specific mass feature 202 at a retention index of 1784. The compound is present at similar levels in all *Nt*-AO lines. Both compounds are absent from the wild-type sample. In addition to the chromatograms, the characteristic mass spectrum (m/z) of each compound is shown for the standard and for one of the artemisinic acid operon lines. EPY224: yeast strain that produces amorpha-4,11-diene (Ro et al., 2006). One representative plant per line is depicted. Mass spectra and mass features of trimethylsilylated artemisinic alcohol are shown. **DOI:** http://dx.doi.org/10.7554/eLife.13664.016

**Figure 7.**
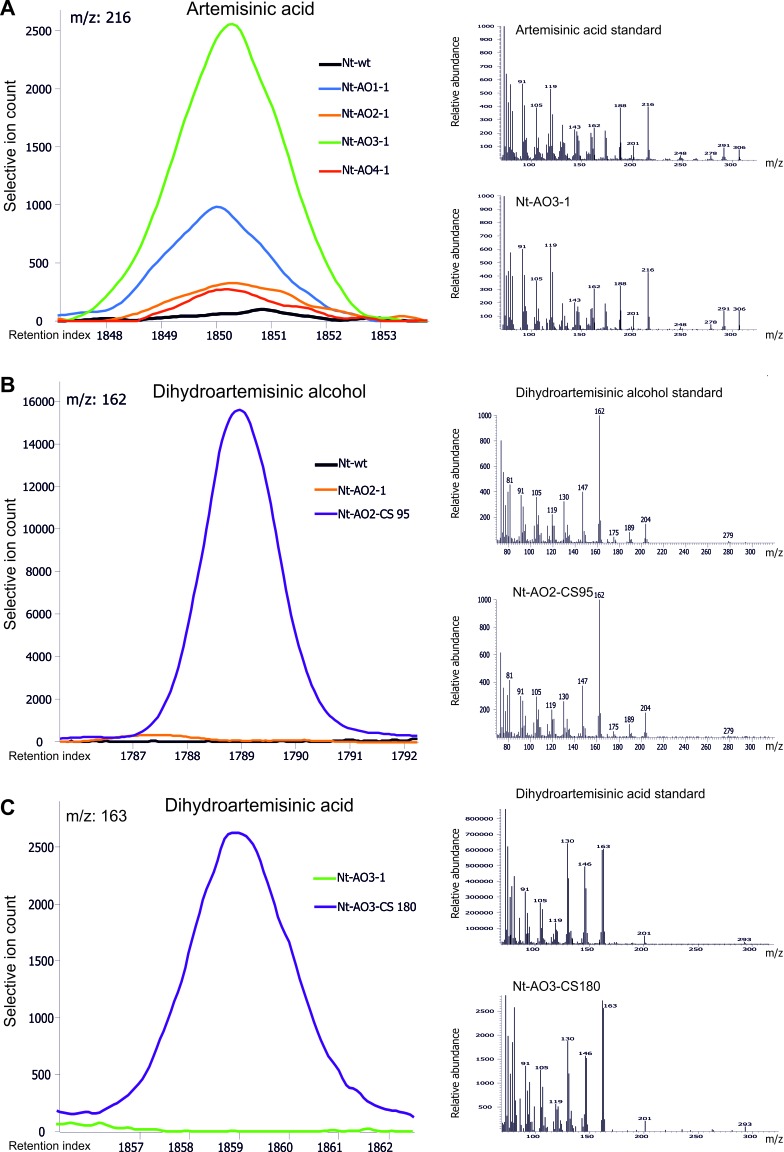
Chromatograms and mass spectra of the artemisinic compounds artemisinic acid, dihydroartemisinic alcohol and dihydroartemisinic acid. Characteristic peaks for one specific fragment at the expected retention index are shown for each compound. (**A**) Artemisinic acid-specific mass feature 216 is shown at a retention index of 1850. This compound accumulates to higher levels in lines *Nt*-AO1-1 and *Nt*-AO3-1. (**B**) Dihydroartemisinic alcohol-specific mass feature 162 at a retention index of 1789. The compound is present at high levels in COSTREL line *Nt*-AO2-CS95, but is absent from transplastomic line *Nt*-AO2-1. (**C**) Dihydroartemisinic acid-specific mass feature 163 at a retention index of 1859. This compound accumulates in COSTREL line *Nt*-AO3-CS180, but is absent from transplastomic line *Nt*-AO3-1. All compounds are absent from the wild-type sample. In addition to the chromatograms, the characteristic mass spectrum of each compound is shown for the standard and for one of the artemisinic acid operon lines. Mass spectra and mass features of trimethylsilylated artemisinic compounds are shown. **DOI:** http://dx.doi.org/10.7554/eLife.13664.017

Increased amounts of artemisinic acid in the *Nt*-AO-CS lines were negatively correlated with the accumulation of artemisinic alcohol (Figure 5A,B), indicating that the efficiency of oxidation of the alcohol represents a key bottleneck in the pathway that we alleviated by supertransformation with the additional pathway genes. Importantly, artemisinic alcohol was reduced to nearly undetectable levels in the best-performing line *Nt-*AO3-CS180 suggesting that maximum conversion efficiency has been achieved (Figure 5B; Figure 5—source data 1). Another significant correlation at the metabolite level was a strong positive correlation between amorpha-4,11-diene and artemisinic alcohol (Tables 1 and 2) which may be a consequence of the enzymatic limitation downstream of artemisinic alcohol. By contrast, amorpha-4,11-diene was not significantly correlated with artemisinic acid accumulation. Dihydroartemisinic alcohol (presumably generated by an endogenous enzymatic activity in tobacco; Ting et al., 2013; Zhang et al., 2011), while accumulating in some lines, showed only a weak positive correlation with artemisinic acid in the *Nt*-AO2-CS but not in the *Nt*-AO3-CS lines (Figure 5—figure supplement 1; Tables 1 and 2; Figures 6 and 7). Dihydroartemisinic acid, the direct precursor of artemisinin, was detected only in line *Nt*-AO3-CS180 in the T0 generation and in lines *Nt*-AO3-CS53 and *Nt*-AO3-CS180 in the T1 generation (Figure 5—source data 1 and Figure 5—figure supplement 1; Figure 7C), and therefore had to be excluded from the correlation analysis.

**Table 1.** Correlation analysis of artemisinic compounds and transgenes introduced into transplastomic line *Nt*-AO2-1 by combinatorial supertransformation. The levels of the artemisinic compounds amorpha-4,11-diene, artemisinic alcohol, dihydroartemisinic alcohol and artemisinic acid, and the presence of the transgenes *dxr, CYB5, ADH1, ALDH1* and *DBR2* were correlated using Spearman’s method in the 39 *Nt*-AO2-CS lines analyzed by genomic PCR in the T0 generation (see Figure 5—source data 1) using the SPSS software. Dihydroartemisinic acid was excluded from this analysis, because it was not detectable in any of the *Nt*-AO2-CS lines. CC: correlation coefficient. Positive values indicate positive correlations and negative values indicate negative correlations. *: p<0.05; **: p<0.01; N: number of samples where both variables are present. **DOI:** http://dx.doi.org/10.7554/eLife.13664.014

*Nt*-AO2-CS		Amorpha-4,11-diene	Artemisinic alcohol	Dihydroartemi-sinic alcohol	Artemisinic acid	*dxr*	*CYB5*	*ADH1*	*ALDH1*	*DBR2*
Amorpha-4,11-diene	CC	1.000	0.602^**^	0.147	-0.112	0.067	-0.020	-0.009	-0.015	0.156
	N	35	29	13	24	35	35	35	35	35
Art. alcohol	CC	0.602^**^	1.000	-0.557^*^	-0.141	-0.159	-0.165	-0.170	0.013	-0.217
	N	29	29	13	24	29	29	29	29	29
Dihydroart. alcohol	CC	0.147	-0.557^*^	1.000	0.642^*^	0.462	0.248	0.383	0.180	0.496
	N	13	13	13	13	13	13	13	13	13
Art. acid	CC	-0.112	-0.141	0.642^*^	1.000	0.404	0.159	0.179	0.207	0.317
	N	24	24	13	24	24	24	24	24	24
*dxr*	CC	0.067	-0.159	0.462	0.404	1.000	0.643^**^	0.402^*^	0.546^**^	0.578^**^
	N	35	29	13	24	39	39	39	39	39
*CYB5*	CC	-0.020	-0.165	0.248	0.159	0.643^**^	1.000	0.192	0.507^**^	0.793^**^
	N	35	29	13	24	39	39	39	39	39
*ADH1*	CC	-0.009	-0.170	0.383	0.179	0.402^*^	0.192	1.000	0.372^*^	0.270
	N	35	29	13	24	39	39	39	39	39
*ALDH1*	CC	-0.015	0.013	0.180	0.207	0.546^**^	0.507^**^	0.372^*^	1.000	0.420^**^
	N	35	29	13	24	39	39	39	39	39
*DBR2*	CC	0.156	-0.217	0.496	0.317	0.578^**^	0.793^**^	0.270	0.420^**^	1.000
	N	35	29	13	24	39	39	39	39	39

**Table 2.** Correlation analysis of artemisinic compounds and transgenes introduced into transplastomic line *Nt*-AO3-1 by combinatorial supertransformation. The levels of the artemisinic compounds amorpha-4,11-diene, artemisinic alcohol, dihydroartemisinic alcohol and artemisinic acid, and the presence of the transgenes *dxr, CYB5, ADH1, ALDH1* and *DBR2* were correlated using Spearman’s method in the 61 *Nt*-AO3-CS lines analyzed by genomic PCR in the T0 generation (see Figure 5—source data 1) using the SPSS software. Dihydroartemisinic acid had to be excluded from this analysis, because it was detectable only in one of the *Nt*-AO3-CS lines in the T0 generation. Note that the negative correlation between artemisinic alcohol and artemisinic acid (Figure 5A,B) is restricted to those lines that display increased artemisinic acid contents, and therefore is not statistically significant over all COSTREL lines analyzed (cf. Figure 5—source data 1). CC: correlation coefficient. Positive values indicate positive correlations and negative values indicate negative correlations. *: p<0.05; **: p<0.01; N: number of samples where both variables are present. **DOI:** http://dx.doi.org/10.7554/eLife.13664.015

*Nt*-AO3-CS		Amorpha-4,11-diene	Artemisinic alcohol	Dihydroartemi-sinic alcohol	Artemisinic acid	*dxr*	*CYB5*	*ADH1*	*ALDH1*	*DBR2*
Amorpha-4,11-diene	CC	1.000	0.363^**^	0.079	0.237	0.006	0.082	0.439^**^	-0.032	0.049
	N	60	58	39	59	60	60	60	60	60
Art. alcohol	CC	0.363^**^	1.000	0.124	-0.081	-0.088	-0.119	0.086	0.026	-0.009
	N	58	59	39	59	59	59	59	59	59
Dihydroart. alcohol	CC	0.079	0.124	1.000	0.029	-0.169	0.153	0.306	0.195	0.219
	N	39	39	39	39	39	39	39	39	39
Art. acid	CC	0.237	-0.081	0.029	1.000	0.241	0.183	0.217	0.386^**^	0.141
	N	59	59	39	60	60	60	60	60	60
*dxr*	CC	0.006	-0.088	-0.169	0.241	1.000	0.153	0.224	0.415^**^	0.170
	N	60	59	39	60	61	61	61	61	61
*CYB5*	CC	0.082	-0.119	0.153	0.183	0.153	1.000	0.185	0.322^*^	0.503^**^
	N	60	59	39	60	61	61	61	61	61
*ADH1*	CC	0.439^**^	0.086	0.306	0.217	0.224	0.185	1.000	0.339^**^	0.252^*^
	N	60	59	39	60	61	61	61	61	61
*ALDH1*	CC	-0.032	0.026	0.195	0.386^**^	0.415^**^	0.322^*^	0.339^**^	1.000	0.444^**^
	N	60	59	39	60	61	61	61	61	61
*DBR2*	CC	0.049	-0.009	0.219	0.141	0.170	0.503^**^	0.252^*^	0.444^**^	1.000
	N	60	59	39	60	61	61	61	61	61

To correlate the genotype of the COSTREL lines with their metabolic phenotypes, the transgene sets present in the nucleus of *Nt*-AO2-CS and *Nt*-AO3-CS lines were determined (Figure 5—source data 1). Statistical analysis revealed that elevated artemisinic acid levels in *Nt*-AO3-CS lines were most strongly correlated with the presence of the *ALDH1* transgene. Weaker (and statistically not significant) correlations were observed between high artemisinic acid levels and the presence of *dxr* and *ADH1*, and, in *Nt*-AO2-CS lines, also the *DBR2* and *ALDH1* transgenes (Tables 1 and 2). These results indicate that *ALDH1* and *dxr* are most likely the genes with the greatest impact on the increase in artemisinic acid content. As transgene presence is not necessarily indicative of transgene expression, we measured mRNA accumulation in the T1 generation of a selected set of COSTREL lines by qRT-PCR analyses. The results support the importance of *dxr, ADH1, ALDH1* and *DBR2* in boosting artemisinic acid synthesis and revealed that the best-performing line (*Nt-*AO3-CS180) expresses *dxr, ADH1, ALDH1* and *DBR2* to high levels (Figure 5C).

To test whether artemisinic acid accumulation is correlated with a visible phenotype of the plants, the best-performing COSTREL lines were compared with their transplastomic recipient lines. No significant phenotypic differences were observed and even line *Nt-*AO3-CS180 (that showed the strongest increase in artemisinic acid accumulation; Figure 5A) was nearly indistinguishable from its transplastomic recipient *Nt-*AO3-1 (Figure 5D). This suggests that artemisinic acid is not toxic to plant cells (and that a further increase in artemisinic acid might be achievable). Growth and biomass measurements confirmed that there are no significant differences between transplastomic line *Nt*-AO3-1 and the best-performing COSTREL line *Nt*-AO3-CS180, and revealed only a small reduction in total leaf biomass (by on average 13%) of the COSTREL line compared to the wild type (Figure 5—figure supplement 2).

None of our transplastomic lines and none of the analyzed COSTREL lines accumulated detectable levels of artemisinin (see Materials and Methods). This could be because the set of transgenes introduced into tobacco was insufficient to obtain conversion of artemisinic acid into artemisinin. However, some of our best-performing COSTREL lines accumulated detectable amounts of dihydroartemisinic acid (Figure 5—source data 1 and Figure 5—figure supplement 1; Figure 7), the immediate precursor of artemisinin, indicating that DBR2 and ALDH1 can function in plastids. The chloroplast is likely to produce sufficient amounts of singlet oxygen (which is a regular by-product of photosynthetic electron transfer) to facilitate the spontaneous conversion of dihydroartemisinic acid into artemisinin (Kopetzki et al., 2013; Figure 1). An alternative explanation for the lack of artemisinin accumulation could be that COSTREL lines that produce artemisinin were not recovered, because artemisinin is highly toxic to photosynthetically active cells (Bharati et al., 2012). The fact that no artemisinin could be detected and only low amounts of dihydroartemisinic acid were obtained in a few lines, whereas artemisinic acid accumulated to high amounts, may indicate that future efforts should be focused on maximizing the production of artemisinic acid.

## Discussion

In the course of this work, we have developed a new synthetic biology approach that combines chloroplast transformation with combinatorial nuclear transformation and large-scale metabolic screening of supertransformed plant lines. This strategy enabled the transfer of an entire biochemical pathway of secondary metabolism from a medicinal plant to a high-biomass crop.

For the foreseeable future, ACTs will remain the most powerful weapon in the world’s battle against malaria (http://www.who.int/malaria/areas/treatment/overview/en/). When used as an oral monotherapy, artemisinin can promote the development of resistance in the parasite (Mok et al., 2015; Straimer et al., 2015; Mbengue et al., 2015) and, therefore, ACTs are based on fixed-dose co-formulations that combine two different active ingredients in one tablet. Development of an inexpensive and sustainable production method that is suitable to meet the constantly growing demand for artemisinin and its derivatives has remained a grand challenge. Enormous breeding efforts are currently underway to produce new varieties of *A. annua* that accumulate higher and more consistent levels of the compound (Graham et al., 2010). However, as *A. annua* produces artemisinin only in a very small fraction of the leaf cells (the glandular trichomes) and its cultivation is inefficient, slow and vulnerable to adverse environmental conditions, the development of a production method that is independent of *A. annua* is highly desirable (Bryant et al., 2015). If accomplished in a high-biomass non-food/non-feed crop, this would provide a stable supply of the feedstock that can be scaled up at will and at short notice, and take full advantage of the existing agricultural infrastructure. In the course of this work, we have established tobacco as an efficient production factory for artemisinic acid. Tobacco is a high-biomass crop, grown in large acreages, for which alternative uses (that are unrelated to smoking) have long been sought. Since tobacco is well suited for cultivation at high cropping densities and multiple harvests (4–5) per season are possible, 40 t of biomass can be obtained from a single acre of tobacco field at a cost of only around $100 per ton (http://tobacco.ces.ncsu.edu/wp-content/uploads/2012/07/tobacco-production-cost-2011-1.pdf?fwd=no). Thus, with our best-performing COSTREL line, production levels of ~4.8 kg artemisinic acid per acre can be obtained, suggesting that the current world demand (of ~100 t artemisinin) can be met by cultivating tobacco on an area of ~200 km^2^, which is less than the area of the city of Boston (assuming ~50% loss during extraction and conversion of artemisinic acid to artemisinin; Paddon et al., 2013; Kopetzki et al., 2013).

Whereas in *A. annua* the artemisinin biosynthetic pathway is confined to glandular trichomes, our COSTREL tobacco lines produce artemisinic acid in chloroplasts and, thus, in the whole leaf. Together with the absence of toxic effects of artemisinic acid on the chloroplast (Figure 5D; Figure 5—figure supplement 2), this offers great potential for further enhancement of the pathway by addressing the bottlenecks that limit flux in our current best-performing lines. Furthermore, previous transgenic work has shown that the redox environment in the cytosol of tobacco cells favors reduction of aldehydes to alcohols rather than their oxidation to acids, thus limiting the ability of the cytosolically located pathway to produce high quantities of artemisinic acid (Zhang et al., 2011). The high levels of artemisinic acid achieved in this work by implementing the pathway into plastids suggest that the chloroplast offers a more favorable redox milieu that allows the quantitative conversion of artemisinic alcohol into artemisinic acid (Figure 5A,B). Although tobacco leaves also possess glandular trichomes (where artemisinin is produced in *A. annua*), the trichomes in our COSTREL plants are unlikely to accumulate large amounts of artemisinic acid. This is because transgene expression from the plastid genome is generally very low in non-photosynthetic tissues and cell types. It can be significantly enhanced by designing specific (chimeric) expression signals that confer high transgene activity in non-green tissues (Zhang et al., 2012; Caroca et al., 2013), but the expression signals used to drive our synthetic artemisinic acid operons (Figure 2) are not suitable to trigger efficient gene expression in non-photosynthetic plastids.

The chloroplast represents an attractive site for engineering new metabolic pathways into plants. Being the biosynthetic center of the plant cell, the chloroplast contains large pools of diverse metabolites that can be tapped. Expression of genes for metabolic enzymes from the plastid genome has a number of attractions, including high expression levels, simple stacking of multiple transgenes in synthetic operons (Lu et al., 2013; Gnanasekaran et al., 2016) and high-precision engineering via homologous recombination (Maliga, 2004; Bock, 2015). Previously, plastid transformation was employed to enhance endogenous metabolic pathways (Apel et al., 2009; Lu et al., 2013) or to produce novel metabolites, such as ketocarotenoids and biopolymers (Hasunuma et al., 2008; Bohmert-Tatarev et al., 2011). Recently, two ER-resident cytochrome P450 enzymes of the dhurrin pathway (a cyanogenic glucoside from sorghum) were successfully expressed from a synthetic operon in tobacco chloroplasts (Gnanasekaran et al., 2016). Together with the third pathway enzyme, a glucosyltransferse, the two P450 enzymes catalyzed the formation of dhurrin from tyrosine. The activity of the P450 enzymes was strictly light-dependent, indicating that the electrons used come from the photosynthetic electron transport chain (Gnanasekaran et al., 2016). This suggests that, at least when P450 enzymes are anchored to the thylakoid membrane, reduced ferredoxin can replace the NADPH-dependent native reductase (Gnanasekaran et al., 2016), thus making the chloroplast a superb compartment for the implementation of secondary metabolic pathways that involve P450-catalyzed reactions.

By transplastomic introduction of the core pathway for artemisinic acid synthesis, our COSTREL approach takes advantage of the stability and high efficiency of transgene expression from the plastid genome (Maliga, 2004; Bock, 2015). Subsequent combinatorial supertransformation of the nuclear genome with genes for auxiliary and regulatory factors then allows fine-tuning of the pathway and optimization of metabolic flux by screening metabolic phenotypes of hundreds of transgenic lines that differ in the set of transgenes they harbor in the genome and the expression levels of the transgenes (Zhu et al., 2008; Naqvi et al., 2009; 2010). Importantly, this approach requires no prior knowledge about the contributions of the individual factors to metabolic flux and the optimum expression strength of each transgene. Previous metabolic engineering work in microorganisms has demonstrated that the success is often more dependent on achieving the optimum balance of enzyme activities than on the absolute levels of enzyme (over)expression (e.g., Peralta-Yahya et al., 2012). The use of combinatorial supertransformation, therefore, provides a significant advantage over the construction of large transformation vectors expressing multiple pathway genes, because the great variation between transgenic events in (i) the transgene combination present, (ii) the copy numbers of the individual transgenes and (iii) the absolute and relative expression strengths of the transgenes (depending, e.g., on the integration site in the genome and the structure of the transgenic locus) is likely to yield at least some events that harbor the optimum combination of transgenes and provide the right balance of enzyme activities. Moreover, the characterization of these elite events can provide valuable information about pathway regulation, limiting steps and bottlenecks that should be the target of future engineering and optimization efforts. In sum, our COSTREL strategy provides a new synthetic biology tool that facilitates the efficient transfer of complex metabolic pathways into new host organisms while, at the same time, maximizing the metabolic output.

## Materials and methods

### Plant material and growth conditions

Tobacco plants (*Nicotiana tabacum* cv. Petit Havana) were grown under sterile conditions on agar-solidified MS medium (Murashige and Skoog, 1962) supplemented with 30 g/L sucrose. Genetically modified plants were selected, propagated and rooted in the same medium containing additionally 500 mg/L spectinomycin (transplastomic plants) or 50 mg/L kanamycin (combinatorially supertransformed plants). For sampling and seed production, plants were transferred to soil and grown under standard greenhouse conditions.

### Construction of transformation vectors

The synthetic operon constructs for chloroplast transformation (pAO1-4) are based on plastid transformation vector pKP9 (Zhou et al., 2008). They all contain the four genes required for the canonical artemisinic acid biosynthetic pathway in *Artemisia annua: FPS* (AF112881), *ADS* (AF138959), *CYP71AV1 (CYP*, DQ268763) and *CPR* (DQ318192; Figure 1). The genes were codon optimized for expression in the chloroplast and chemically synthesized (GeneArt, Regensburg, Germany). The four genes were then assembled into synthetic operons as follows. The *CYP71AV1 (CYP*) gene was synthesized with a Shine-Dalgarno (SD) sequence derived from the chloroplast *rbcL* gene and with the flanking restriction sites NheI (at the 5’ end) and XbaI (at the 3’ end). The gene was cloned into pZF1 replacing the *P24* gene (Zhou et al., 2008) and generating construct pZF83. pZF1 is an intermediate cloning construct that contains the promoter from the rRNA operon from tobacco (P*rrn*), the leader sequence from the *gene 10* of bacteriophage T7 (*T7* L*g10*), the *P24* capsid protein gene of HIV-1 and the terminator of the chloroplast *rbcL* gene (T*rbcL*; Zhou et al., 2008). A fragment containing the rRNA operon promoter from *Chlamydomonas reinhardtii (Cr* P*rrn*), the T7 L*g10*, the *gfp* gene, the terminator of the *atpA* gene from the chloroplast genome of *C. reinhardtii (Cr* T*atpA*) and the intercistronic expression element (IEE; Zhou et al., 2007) was excised with SacI and NheI from a modified version of construct pDK139 in which the ClaI, SalI and XhoI restriction sites between *Cr* T*atpA* and IEE were removed by XhoI/HindIII digestion and blunting of the overhanging ends by a fill-in reaction with Klenow enzyme. pDK139 is a chloroplast transformation construct based on vector pHK20 (Kuroda and Maliga, 2001). The excised fragment was cloned into pZF83, replacing the region spanning P*rrn* and T7 L*g10* and generating construct pZF84. Next, the *FPS* gene was synthesized flanked by NdeI and PacI restriction sites at the 5’ and 3’ ends, respectively. The excised NdeI/PacI restriction fragment was cloned into the identically digested pZF84, replacing the *gfp* gene and giving rise to plasmid pZF85. The complete fragment from *Cr* P*rrn* to T*rbcL* was then cut out from pZF85 with SacI and ClaI and ligated into chloroplast transformation vector pKP9 (Zhou et al., 2008), producing clone pZF90. The *ADS* gene was synthesized (flanked by NcoI and EcoRV restriction sites) and cloned into vector pKCZaphA-6, replacing the *aphA-6* gene and giving rise to plasmid pZF86. pKCZaphA-6 (Fleischmann et al., 2011) is an intermediate cloning construct that contains the *C. reinhardtii psbA* promoter (*Cr* P*psbA*), the *C. reinhardtii psbA* leader (*Cr* L*psbA*), the *aphA-6* gene for kanamycin resistance and the *C. reinhardtii rbcL* terminator (*Cr* T*rbcL*). Next, the terminator of the tobacco *rps16* gene (T*rps16*) was amplified by PCR with primers containing EcoRV and PstI restriction sites at the 5’ and 3’ ends, respectively, and cloned into pZF86 digested with the same enzymes, generating vector pZF87. The *CPR* gene was synthesized as a PstI/SphI restriction fragment with the *rbcL* SD sequence and an IEE element at its 5’ end. The fragment was cloned into pZF87 digested with the same enzymes, giving rise to pZF88. Artemisinic acid operon constructs pAO1 and pAO2 were generated by digesting pZF88 with ClaI (releasing the cassette containing the *ADS-CPR* dicistron between *Cr* P*psbA* and *Cr* T*rbcL*) and cloning this cassette into pZF90 digested with the same enzyme. In vector pAO1, the *ADS-CPR* cassette is integrated in sense orientation, downstream of the *FPS-CYP* cassette, whereas in construct pAO2, the fragment is integrated in antisense (Figure 2A). For generation of pAO3 and pAO4, the *Cr* P*psbA - Cr* L*psbA* fragment was eliminated from pZF88 by digestion with MluI and NcoI and subsequently replaced by a PCR-amplified *Cr* P*psbA* - T7 L*g10* fragment obtained by digestion with the same enzymes, thus generating plasmid clone pZF89. pZF89 was then digested with ClaI and cloned into pZF90 in a similar way as for generation of pAO1 and pAO2. Construct pAO3 originates from integration of the *ADS-CPR* cassette in sense orientation, whereas pAO4 harbors the cassette in antisense orientation (Figure 2A).

Constructs pCS1-5 for combinatorial supertransformation contain the genes *dxr* (BA000022) from *Synechocystis* sp. and *CYB5* (JQ582841.1), *ADH1* (JF910157.1), *ALDH1* (FJ809784.1) and *DBR2* (EU704257.1) from *A. annua*. The genes were codon optimized for expression in the nucleus and synthesized (Eurofins MWG Operon). The five constructs are derivatives of pUC18 and contain the terminator from the nopaline synthase gene (T*nos*), the transit peptide from *RBCS* and either the *35S* promoter from the cauliflower mosaic virus (CaMV), the mannopine synthase gene promoter from *Agrobacterium tumefaciens* (P*mas*) or the ubiquitin-10 promoter from *Arabidopsis thaliana* (P*UBIQ10*). To generate these constructs, the *RBCS* transit peptide (TP) was amplified by PCR with primers introducing XbaI/XhoI, ApaI/XhoI or SpeI/XhoI restriction sites into the 5’ and 3’ ends of the amplification product, respectively. The TP was then digested with the corresponding restriction enzymes and cloned into a P*35S*-T*nos* cassette (opened with XbaI/XhoI), a P*mas*-T*nos* cassette (opened with ApaI/XhoI) and a P*UBIQ10*-T*nos* cassette (opened with SpeI/XhoI), producing constructs pPF28, pPF29 and pPF30, respectively. Constructs pCS1 and pCS2 are derivatives of pPF28 and were generated by cloning the synthetic genes *dxr* and *CYB5* into pPF28 as XhoI/SacI fragments. Constructs pCS4 and pCS5 are derivatives of pPF29 and were obtained by cloning the synthetic genes *ALDH1* and *DBR2* into pPF29 as XhoI/SacI fragments. Finally, construct pCS3 was obtained in a similar way, by cloning the synthetic gene *ADH1* into pPF30 as an XhoI/XmaI restriction fragment.

The plasmid cocktail for combinatorial transformation was produced by mixing equal quantities of constructs pCS1-5 (each at a concentration of 2 µg/µL) and plasmid pPH200 that contains the *nptII* gene for kanamycin resistance between the CaMV 35S promoter and terminator.

### Plastid transformation and selection of transplastomic tobacco plants

For chloroplast transformation, young leaves harvested from aseptically grown wild-type tobacco plants were bombarded with gold particles covered with plasmid-DNA (pAO1-4) using the DuPont PDS1000He biolistic gun. Spectinomycin-resistant shoots were selected on plant regeneration medium with 500 mg/L spectinomycin (Svab and Maliga, 1993). Primary transformants were identified by Southern blot analysis and at least one additional regeneration round was performed to obtain homoplasmic plants. Independently generated transplastomic lines are designated by the construct number followed by the number of the individual line (e.g., *Nt*-AO1-2 stands for *Nicotiana tabacum* plant obtained with construct pAO1, transplastomic line number 2). Homoplasmy was confirmed by Southern blot analyses and seed assays.

### Combinatorial nuclear supertransformation and selection of transgenic tobacco plants

Young leaves from transplastomic plants *Nt*-AO2-1 and *Nt*-AO3-1 grown under aseptic conditions were harvested and bombarded with gold particles coated with a plasmid DNA mixture containing pCS1-5 and pPH200 using the DuPont PDS1000He biolistic gun. Kanamycin-resistant shoots were selected on plant regeneration medium containing 50 mg/L kanamycin. Resistant shoots were rooted in the same medium, then transferred to soil and grown to maturity under standard greenhouse conditions. Material from T0 plants was used for initial molecular analyses and preliminary metabolite profiling experiments. To generate standardized material for metabolite measurements and molecular analysis of the T1 generation, seeds from candidate supertransformed lines were surface-sterilized and sown on MS medium with 200 mg/L kanamycin. After three weeks, six green (resistant) seedlings per line were transferred to soil and raised under standard greenhouse conditions.

### Plant growth and biomass measurements

Plant height and total leaf biomass were determined for six plants each of *N. tabacum* wild type (wt), the transplastomic line *Nt*-AO3-1 and the progeny of four *Nt*-AO3-CS180 T1 lines. Measurements were performed at two different stages. The first measurement was done when the wild-type plants started to flower ('same age'). The second measurement was done when the *Nt*-AO3-1 and *Nt*-AO3-CS180 plants started to flower (typically five days after the first measurement), to compensate for the slightly delayed development of the transplastomic plants and the COSTREL plants. The height was measured from the top of the pot to the top of the inflorescence. The total leaf biomass (fresh weight, FW) was determined by weighing all leaves of an individual plant.

### Isolation of nucleic acids

Total plant DNA was extracted from frozen leaf material by a CTAB-based protocol (Doyle and Doyle, 1990). For total RNA extraction, samples of 300–400 mg of frozen powdered plant material were extracted with the peqGOLD Trifast reagent (Peqlab GmbH, Erlangen, Germany), following the manufacturer’s instructions. The RNA pellet was resuspended in 100 µL of RNase-free water and mixed with 250 µL buffer RA1 from the NucleoSpin RNA Plant kit (Macherey-Nagel, Düren, Germany). 350 µL of 70% EtOH were mixed with the RNA solution, passed through the RNA-binding column and purified following the protocol of the supplier. Finally, the RNA was eluted in 45 µL of RNase-free water and stored at -80°C until use.

### cDNA synthesis

Prior to reverse transcription, isolated RNAs were tested for the presence of contaminating DNA by a standard PCR using 1 ng of RNA as template. If no DNA amplification was observed, cDNA was synthesized as follows. 1.5 µg of RNA were incubated with 1 µL of oligo(dT) primer (10 µM) and 1 µL of dNTPs (10 mM) for 5 min at 65°C. Then, 7 µL of a master mix were added (4 µL of 5x First Strand buffer, 1 µL 0.1 M DTT, 40 U RNaseOUT and 200 U SuperScript III Reverse Transcriptase; Invitrogen, Carlsbad, CA) and incubated for 1 hr at 50°C, followed by an inactivation step of 15 min at 70°C. Alternatively, cDNA was synthesized using the QuantiTect Reverse Transcription kit (Qiagen, Hilden, Germany) following the manufacturer's instructions. The quality of the cDNA was tested by standard PCR.

### Quantitative real-time PCR (qRT-PCR)

Quantitative RT-PCR was performed in a LightCycler 480 (Roche, Mannheim, Germany) using cDNA as template in 5 µL reactions containing 1 µL of each gene-specific primer (1.25 µM; Table 3), 2.5 µL of the LightCycler 480 SYBR green I Master mix and 0.5 µL of a 1:50 cDNA dilution. Three biological (independent plants) and three technical replicates per line were analyzed. The relative transcript levels were determined using the formula (1+E)^-ΔΔCt^ where E is the binding efficiency of the primers (Pfaffl, 2001). E was calculated from the slope of the expression level of each gene in a dilution series of a given cDNA. Results were normalized to the mRNA levels of *ACTIN* as a housekeeping gene (Table 3), and relative mRNA accumulation levels were calculated according to the delta-delta *C*t method. To identify the key genes involved in the increased levels of artemisinic acid in supertransformed plants, the expression levels of each transgene (in all lines were it was present) were compared by One-way ANOVA analysis (p<0.05). The results were expressed as a heat map, where the darkest green color represents the highest expression level (brown: no expression).

**Table 3.** List of oligonucleotides used in this study. The reverse primers (_R) for amplification of the genes *FPS, ADS, CYP* and *CPR* contain the sequence of the T7 promoter (bold) to facilitate in vitro transcription. **DOI:** http://dx.doi.org/10.7554/eLife.13664.018

Gene	Primer	Sequence (5'→3')	Purpose
*dxr*	DXR_F	CAACCTATGTACGTTGTTGGAGAAGAGGG	qPCR
	DXR_R	CTGGAGCACCAGCAATCAATGTCTC	
*CYB5*	CYB5_F	CCAGGAGGAGATGAAGTTCTTTTGGCTG	qPCR
	CYB5_R	GCTGGAGGAACGTAAGCTCTCTTCTTTG	
*ADH1*	qADH1_F2	TCCAGGTCATGAAGGTGTTG	qPCR
	qADH1_R2	ATTGTCCACACTCACCAAGG	
*ALDH1*	ALDH1_F	CCTGTTTCTTTGGAATTGGGTGGTAAGTC	qPCR
	ALDH1_R	CAGCAACACACATCTCACCTTTGTTAGTG	
*DBR2*	DBR2_F	GAGCAAGTTGAGGGTTGGAAGAAAGTTG	qPCR
	DBR2_R	TAGAAGAGATAGGAGCAGCTCCACCTG	
*CYP*	CYP_qF	CCTGAACCTTGGAGATTACC	qPCR
	CYP_qR	GCCCATTTAGGAGAAGATACAAC	
*CPR*	CPR_qF	CCTGTTGGAATGGGTGATG	qPCR
	CPR_qR	CCTACAGCAGCAGTATAAGGAG	
*ACTIN*	qTac9actin f	CCTGAGGTCCTTTTCCAACCA	qPCR
	qTac9actin r	GGATTCCGGCAGCTTCCATT	
*FPS*	FPS_probe_F	CCTGCTTTTGAATTTGATGATG	RNA probe
	FPS_probe_R	**TAATACGACTCACTATAGGG**CGAAACCAACAAGGTTGTCC	
*ADS*	ADS_probe_F	CTGAAGCTGTTGAAAGATGGTC	RNA probe
	ADS_probe_R	**TAATACGACTCACTATAGGG**GGAGCAGATACAGCCCATTC	
*CYP*	CYP_probe_F	TCCTCATCGAGGAGTACGAG	RNA probe
	CYP_probe_R	**TAATACGACTCACTATAGGG**TTACAGGTCGTCCAGATCCAG	
*CPR*	CPR_probe_F	ATGATTGGTCCTGGAACTGG	RNA probe
	CPR_probe_R	**TAATACGACTCACTATAGGG**GCCATTCCTTTAGCATCTCC	

### Synthesis of hybridization probes and gel blot hybridizations

For Southern blot analysis, samples of 2–3 µg DNA were digested with BamHI, separated by electrophoresis in 0.8% agarose gels and transferred onto Hybond XL nylon membranes (GE Healthcare, Little Chalfont, UK) by capillary blotting. For northern blot analysis, samples of 4–5 µg total RNA were separated in denaturing formaldehyde-containing agarose gels (1.5%) and transferred onto nylon membranes. As RFLP probe, a 550-bp fragment of the *psaB* gene was amplified by PCR using primer pair P7247 / P7244 (Wurbs et al., 2007) and purified. The probe was labeled with [α^32^P]dCTP by random priming (Multiprime DNA labeling kit; GE Healthcare). Probes for *FPS, ADS, CYP* and *CPR* were generated by in vitro transcription and radioactive labeling with [α^32^P]UTP. PCR fragments of 200–300 bp were amplified for each gene using specific primers (Table 3) that contain the T7 promoter sequence in the reverse primer. Radiolabeled probes were generated by incubating 5 µL PCR product with 4 µL H_2_O, 2 µL 10x buffer, 3 µL of an equimolar mixture of ATP, CTP and GTP, 2 µL T7 RNA polymerase (15 U/µL) and 4 µL [α^32^P]UTP (40 µCi) for 30 min at 37°C. Hybridizations were performed overnight at 65°C. Following standard washing steps, autoradiographic screens were exposed to the membranes for 3–4 hr and then scanned in a Typhoon TRIO+ scanner (GE Healthcare).

### Cultivation of yeast reference strains

The following genetically engineered strains of *Saccharomyces cerevisiae* were used as reference strains for artemisinic metabolites (Ro et al., 2008): (i) EPY300 (MATαα his3Δ1 leu2Δ0 lys2Δ0 ura3Δ0 PGAL1-tHMGR PGAL1-upc2-1 erg9::PMET3-ERG9 PGAL1-tHMGR PGAL1-ERG20; Ro et al., 2008), a control strain that does not produce artemisinic compounds, (ii) EPY224 (EPY300 transformed with plasmid pRS425-Leu::ADS; Ro et al., 2006), a strain that produces amorpha-4,11-diene, and (iii) EPY302 (EPY300 transformed with plasmids pRS425-Leu::ADS and pESC-Ura::AMO/CPR; Ro et al., 2006), a strain that produces artemisinic acid and artemisinic acid pathway intermediates. Plasmid pRS425-Leu::ADS complements the leucine auxotrophy and plasmid pESC-Ura::AMO/CPR complements the uracil auxotrophy of strain EPY300. The three yeast strains were kindly provided by Dr. Jay D. Keasling (UC Berkley, USA). Yeast strains were maintained on solid synthetically defined (SD) medium supplemented with 2% (w/v) sucrose and 0.002% (w/v) uracil for EPY224, and additionally 0.01% (w/v) leucine for EPY300. Induction of the synthesis of artemisinic compounds in strains EPY224 and EPY302 was done by adding 1.8% (w/v) galactose and 1 mM methionine to liquid SD medium and reducing the sucrose content to 0.2% (w/v). Yeast strains were incubated for 120 hr at 30°C and 160 rpm, until they reached an OD_600_ of 1.4. Control strain EPY300 was incubated under the same conditions in liquid SD medium supplemented with 2% (w/v) sucrose, 0.002% (w/v) uracil and 0.01% (w/v) leucine.

### GC-MS analyses

For GC-MS profiling of volatile organic compounds (VOCs), leaves of *N. tabacum* plants were collected, immediately frozen in liquid nitrogen and processed in a cryogenic grinding robot (Labman, North Yorkshire, UK). Aliquots of 500 ± 10 mg of frozen powdered leaf tissue were weighed in frozen microcentrifuge tubes, and then transferred to frozen 20 mL head-space screw cap vials. The powdered plant material was kept at 15°C in the closed vials for at least 1 hr and then incubated for 10 min at 50°C prior to VOC analysis. VOCs were sampled in a replicated randomized block sequence design by solid phase micro extraction (SPME) using a StableFlex™ SPME fiber with 65 μm polydimethylsiloxane/divinylbenzene coating (Supelco, Bellefonte, USA), and profiled as described previously (Agudelo-Romero et al., 2015; 2013) using a DB-624 capillary column of 60 m length, 0.25 mm internal diameter and 1.40 µm film thickness (Agilent Technologies Deutschland GmbH, Waldbronn, Germany). VOCs were analyzed by gas chromatography coupled to electron impact ionization/quadrupole mass spectrometry (GC-EI/QUAD-MS) using an Agilent 6890N24 gas chromatograph connected to an Agilent 5975B VL mass spectrometer (Agilent Technologies, Böblingen, Germany). Data files were visually controlled, exported in NetCDF file format and baseline-corrected using the Agilent ChemStation software and the MetAlign software (Lommen, 2009). Data processing into a standardized numerical data matrix and compound identification were performed using the TagFinder software (Luedemann et al., 2008). Criteria for manually supervised metabolite identification were the presence of at least three specific and selective mass fragments and a retention time deviation <1.0%. The relative accumulation of amorpha-4,11-diene in VOC profiles of leaf tissue was analyzed using the mass spectral intensity of specific and selective mass fragments (Response) after normalization to fresh weight (Response/FW).

Amorpha-4,11-diene was identified in VOC profiles with the help of the reference substance obtained from cultures of the genetically engineered yeast strain EPY224 (Ro et al., 2006). To this end, the strain was grown in 25 mL of inducing SD medium for 120 hr at 30°C under vigorous shaking (160 rpm), until an OD_600_ of ~1.4 was reached. Additionally, control strain EPY300 was grown in 25 mL of SD medium supplemented with 2% (w/v) sucrose, 0.002% (w/v) uracil and 0.01% (w/v) leucine under the same conditions. Amorpha-4,11-diene was identified by differential display of 1 mL cell suspensions in 20 mL head-space screw cap vials comparing the VOC profiles of the compounds obtained from strain EPY224 with those from control strain EPY300, and analysis of the main differential VOC. VOC profiles obtained from leaf material of *A. annua* were used to further validate the identification of amorpha-4,11-diene. Amorpha-4,11-diene present in the VOCs of tobacco leaf material from transplastomic and combinatorially supertransformed lines was annotated by mass spectral (m/z) and retention time matching to the reference data in the Golm Metabolome Database (GMD, http://gmd.mpimp-golm.mpg.de/; Kopka et al., 2005). For compound information and reference data, see the GMD entry for amorpha-4,11-diene (GMD identifier: A149010; http://gmd.mpimp-golm.mpg.de/search.aspx). The retention time of amorpha-4,11-diene in the VOC analysis (Agudelo-Romero et al., 2015) of tobacco plant samples was on average 1563 s, with less than 1% deviation between independent experiments. The specific fragments used for verification of the identity of the compound in complex samples were m/z 93, 105, 119, 133, 189 and 204 (Figure 6A).

For preparation and GC-MS profiling of lipophilic saponification products from total leaf tissue containing artemisinic acid and/or intermediates of artemisinic acid biosynthesis, aliquots of 150 ± 5 mg of frozen powdered *N. tabacum* leaves were mixed with 500 µL of 2 N KOH/methanol, and incubated at 70°C for 1 hr with gentle shaking (at 800 rpm). After acidification of the saponified samples with 100 µL of 12 M HCl, 300 µL of hexane were added and the samples were vortexed for 1 min. After centrifugation for 5 min at 14,000 rpm, 200 µL of the hexane extract were transferred into a clean microcentrifuge tube and concentrated under a mild N_2_ flow to near dryness. Samples were manually trimethylsilylated. Trimethylsilylation was performed by adding 50 μL of a mixture of N,O-bis(trimethylsilyl)trifluoroacetamide (BSTFA) and an n-alkane standard in hexane (7:1, v/v) followed by incubation at 37°C for 30 min with gentle shaking (800 rpm). Metabolite profiling was performed as detailed previously (Erban et al., 2007) by gas chromatography coupled to electron impact ionization/time-of-flight mass spectrometry (GC-EI/TOF-MS) using an Agilent 6890N24 gas chromatograph (Agilent Technologies) connected to a Pegasus III time-of-flight mass spectrometer (LECO Instrumente GmbH, Mönchengladbach, Germany). Retention indices were calibrated in the range relevant for the intermediates of artemisinic acid biosynthesis by addition of a C_15_/C_18_/C_19_ alkane reference mixture to each sample (Strehmel et al., 2008). Chromatograms were acquired, visually controlled, baseline-corrected and exported in NetCDF file format using the ChromaTOF software (Version 4.22; LECO, St. Joseph, USA). Data analysis of GC-EI/TOF-MS profiles of lipophilic saponification products was performed as described for the VOC analysis. Relative quantification of the intermediates of artemisinic acid biosynthesis was performed by calculating normalized responses/FW values using the response of the C_18_ n-alkane and the fresh weight of the sample.

Intermediates of artemisinic acid biosynthesis were initially identified by comparing GC-EI/TOF-MS profiles from yeast strain EPY224 (synthesizing amorpha-4,11-diene) to those of yeast strain EPY302 (synthesizing artemisinic acid and also accumulating all pathway intermediates) and control strain EPY300 (that does not express any of the pathway enzymes). Strain EPY302 was cultured in the same way as strain EPY224, but without addition of uracil. All extractions were performed in duplicate omitting saponification. One of the two sample sets was trimethylsilylated as described above, while the other sample set remained non-derivatized. Non-derivatized samples from yeast were compared to trimethylsilylated samples to unambiguously link the non-derivatized soluble metabolic intermediates of artemisinic acid (A188031) and artemisinic alcohol (A177023) to their respective trimethylsilylated analytes (artemisinic acid 1TMS, A185023; artemisinic alcohol 1TMS, A178029). Dihydroartemisinic alcohol 1TMS (A179026) and dihydroartemisinic acid 1TMS (A186033) were identified after trimethylsilylation. To further validate the identity of the artemisinic compounds, the trimethylsilylated and non-derivatized GC-EI/TOF-MS profiles from yeast were compared to equivalently processed leaf material of *A. annua*. Finally, GC-EI/TOF-MS profiles from authenticated reference compounds (kindly provided by Andreas Pallidis and Dr. Alexander R. van der Krol, Wageningen University, The Netherlands) were used to unambiguously confirm identification of artemisinic acid (AA), artemisinic aldehyde (AAA), artemisinic alcohol (AAOH), dihydroartemisinic acid (DHAA), dihydroartemisinic aldehyde (DHAAA) and dihydroartemisinic alcohol (DHAAOH). Artemisinic alcohol, dihydroartemisinic alcohol, dihydroartemisinic acid and artemisinic acid were identified as trimethylsilylated chemical derivatives in complex profiles according to their mass spectrum (m/z) and retention time index relative to the C_15_/C_18_/C_19_ n-alkanes, using reference data from the Golm Metabolome Database. Guidelines for manually supervised metabolite identification were the presence of at least 3 specific mass fragments per compound and a retention index deviation <1.0% (Strehmel et al., 2008). The average retention index of artemisinic alcohol (1TMS) was 1785 and the specific fragments used for verification were m/z 91, 105, 119, 132, 162, 187 and 202. The average retention index of dihydroartemisinic alcohol (1TMS) was 1789 and the specific fragments used for verification were m/z 91, 105, 162, 189 and 204. The average retention index of dihydroartemisinic acid (1TMS) was 1859 and the specific fragments used for verification were m/z 91, 105, 119, 130, 162, 163, 293 and 308. The average retention index of artemisinic acid (1TMS) was 1851 and the specific fragments used for verification were m/z 91, 105, 119, 188, 216, 291 and 306 (Figures 6 and 7). Retention indices of each compound showed a deviation of less than 1% in all measurements performed. For quantification purposes, the most abundant and specific among the selective mass features of each artemisinic metabolite was chosen, i.e., m/z 162 or 202 for artemisinic alcohol (Figure 6B), m/z 162 or 204 for dihydroartemisinic alcohol (Figure 7B), m/z 163 for dihydroartemisinic acid (Figure 7C), and m/z 188 or 216 for artemisinic acid (Figure 7A).

For absolute quantification of artemisinic acid, we first determined the percentage of recovery of artemisinic acid spiked into wild-type tobacco leaf tissue samples in comparison to the recovery of pure artemisinic acid processed without saponification and in the absence of leaf material. To this end, 150 ± 5 mg of powdered frozen leaf material from *N. tabacum* was mixed with 10 µL of an artemisinic acid standard of known concentration (2 mg/mL in methanol) and subjected to the saponification protocol. The matrix-free artemisinic acid standard was prepared by dissolving 2 mg of artemisinic acid powder (Apin Chemicals, Oxon, UK) in 1 mL of methanol. All spiked samples were prepared and measured in six replicates and compared to the non-saponified matrix-free artemisinic acid reference samples. The average of the artemisinic acid response values obtained from the reference samples was set to 100%, and the percentage of recovery of artemisinic acid from the leaf tissue matrix after saponification was calculated to be 66 ± 16%. This value was used to correct for the final amount of total artemisinic acid in saponified extracts from plant samples. The artemisinic acid concentration in transplastomic and combinatorially supertransformed plants was calibrated using a dilution series of the commercial non-saponified standard. GC-EI/TOF-MS analysis was as described above. The final quantification of artemisinic acid in line *Nt*-AO3-CS180 was done as described above except that, due to the high amounts, only 1/10 of the standard extract volume was used.

For identification of artemisinin or degradation products of artemisinin, aliquots of 1.2 ± 0.01 g of frozen powdered leaf tissue were placed in 20 mL head-space screw cap vials, mixed with 3.6 mL hexane and incubated for 1 hr in a water bath at 69°C. The tubes were shortly vortexed and opened every 10 min to release the vapor pressure. Samples were then centrifuged for 5 min at 14,000 rpm. 300 µL of the hexane extracts were transferred to 1.1 mL Chromacol vials and reduced to 50 µL under a mild flow of N_2_. For identification of artemisinin or its degradation products, 500, 1000 or 2500 ng of an artemisinin standard (1 mg/mL; Sigma-Aldrich, Steinheim, Germany) were subjected to the same procedure. GC-EI/TOF-MS profiling was performed as described for soluble metabolites using the whole tissue saponification protocol. As reported previously, only the degradation products of artemisinin (peaks A and B; Sipahimalani et al., 1991), were detected, likely due to thermal instability of artemisinin. Peaks A and B were only detected in samples that contained the artemisinin reference compound, but not in any of the plant samples.

### UPLC analysis of isoprenoids

For UPLC analysis of pigments, samples of 40 ± 2 mg of frozen powdered leaf tissue were extracted with 500 µL HPLC grade acetone. A stainless steel ball was added to the mixture and the samples incubated for 20 min at 30°C and 1,400 rpm in the dark. After centrifugation for 5 min at 12,000 rpm and 4°C, the upper phase was collected in a new microcentrifuge tube and stored on ice in darkness. The acetone extraction was repeated two more times, using 250 µL of acetone each time and combining the three upper phases. Following centrifugation for 5 min at 12,000 rpm and 4°C to precipitate any remaining insoluble material, 600 µL of the acetone extracts were transferred to 9 mm glass vials. Samples were analyzed using a Waters UPLC Class H (Milford, USA) equipped with an autosampler, Quaternary Solvent Manager, and eλ PDA detector. Pigments were separated in a Waters ACQUITY UPLC BEH C_18_ 1.7 µm C18 2.1 × 50 mm column at 28°C, using UPLC solutions A and B. Elution was carried out at a flow rate of 0.5 mL/min with the following gradient: 100–0% of solution A from 0 to 5 min, 100% solution B from 5 to 6 min, 0–100% solution A from 6 to 6.5 min, and 100% solution A from 6.5 to 7.5 min. Carotenoids were detected at 450 nm and chlorophylls at 640 nm. Three biological replicates (i.e., independent plants) per condition were measured and data were analyzed with the Empower 3 software.
