# Supplementary material for: A new synthetic biology approach allows transfer of an entire metabolic pathway from a medicinal plant to a biomass crop
Source: eLife. 2016 Jun 14;5:e13664. doi: 10.7554/eLife.13664 (PMC4907697; doi:10.7554/eLife.13664)
Supplement: Figure 5—source data 1. — The supertransformed lines Nt-AO2-CS and Nt-AO3-CS are arranged according to their artemisinic acid content, from low to high. Fresh weight corrected response values (R/FW) for amorpha-4,11-diene were multiplied by 1,000 and expressed as Rx1000/FW. Dihydroartemisinic acid was only detectable in line Nt-AO3-CS180 at a low level of 0.03 R/FW. Asterisks mark the selected candidate lines further analyzed in the T1 generation. Lines were clustered according to artemisinic acid content using hierarchical cluster analysis based on Ward’s method. Cnd: cluster nd (artemisinic acid not detected); C1: cluster 1; C2: cluster 2; C3: cluster 3; C4: cluster 4; C5: cluster 5. Genes detected in the genomic PCR assays are numbered as follows: 1: dxr; 2: CYB5; 3: ADH1; 4: ALDH1; 5: DBR2; 0: no gene detected; ?: unclear result. nd: not detected; nm: not measured; -: not determined. Amorpha-4,11-diene values are from one measurement per line, values for artemisinic alcohol, dihydroartemisinic alcohol and artemisinic acid represent averages of three technical replicates per line. SD: standard deviation. DOI: http://dx.doi.org/10.7554/eLife.13664.011 [file elife-13664-fig5-data1.zip › Figure 5_S1.rtf]

Figure 5 - figure supplement 1. Metabolic screening (phenotyping) and genotyping of the T0 generation of combinatorially supertransformed Nt-AO-CS lines. The supertransformed lines Nt-AO2-CS and Nt-AO3-CS are arranged according to their artemisinic acid content, from low to high. Fresh weight corrected response values (R/FW) for amorpha-4,11-diene were multiplied by 1,000 and expressed as Rx1000/FW. Dihydroartemisinic acid was only detectable in line Nt-AO3-CS180 at a low level of 0.03 R/FW. Asterisks mark the selected candidate lines further analyzed in the T1 generation. Lines were clustered according to artemisinic acid content using hierarchical cluster analysis based on Ward's method. Cnd: cluster nd (artemisinic acid not detected); C1: cluster 1; C2: cluster 2; C3: cluster 3; C4: cluster 4; C5: cluster 5. Genes detected in the genomic PCR assays are numbered as follows: 1: dxr; 2: CYB5; 3: ADH1; 4: ALDH1; 5: DBR2; 0: no gene detected; ?: unclear result. nd: not detected; nm: not measured; -: not determined. Amorpha-4,11-diene values are from one measurement per line, values for artemisinic alcohol, dihydroartemisinic alcohol and artemisinic acid represent averages of three technical replicates per line. SD: standard deviation.


Recipient line	Line Nr.	Amorpha-4,11-diene (Rx1000/FW)	Artemisinic alcohol (R/FW)	SD	Dihydro-artemisinic alcohol (R/FW)	SD	Artemisinic acid (µg/g FW)	SD	Genomic PCR	
AO2-CS	48	nd	nd	-	nd	-	ndCnd	-	 	
	176	nd	nd	-	nd	-	ndCnd	-	1,2,3,5	
	326	nd	nd	-	nd	-	ndCnd	-	1,2,3,4,5	
	410	nd	nd	-	nd	-	ndCnd	-	1,2,3,4,5	
	86	nd	nd	-	nd	-	ndCnd	-	0	
	352	0.01	nd	-	nd	-	ndCnd	-	0	
	112	0.01	nd	-	nd	-	ndCnd	-	1,2,3,4,5	
	272	0.02	nd	-	nd	-	ndCnd	-	 	
	1	0.03	nd	-	nd	-	ndCnd	-	 	
	218	0.04	nd	-	nd	-	ndCnd	-	1,3,4	
	32	0.05	nd	-	nd	-	ndCnd	-	1,2,4	
	119	0.05	nd	-	nd	-	ndCnd	-	 	
	180	0.05	nd	-	nd	-	ndCnd	-	0	
	192	0.06	nd	-	nd	-	ndCnd	-	1,3,4	
	6	0.12	nd	-	nd	-	ndCnd	-	 	
	265	0.21	0.03	0.00	nd	-	ndCnd	-	0	
	284	0.53	0.03	0.00	nd	-	ndCnd	-	1,2,3,4,5	
	226	0.99	0.05	0.01	nd	-	ndCnd	-	1	
	19	0.21	0.09	0.00	nd	-	ndCnd	-	1,2,4	
	251	2.69	0.11	0.04	nd	-	ndCnd	-	 	
	143	3.34	0.23	0.02	nd	-	ndCnd	-	1,3?	
	15	2.76	0.25	0.01	0.02	0.00	ndCnd	-	 	
	355	0.98	0.06	0.00	nd	-	1.62C1	0.06	 	
	70	2.06	0.26	0.03	0.02	0.00	1.91 C1	0.08	 	
	172	1.21	0.12	0.02	nd	-	2.33 C1	0.01	 	
	50	0.25	0.05	0.01	nd	-	2.35 C1	0.00	1,2,4,5	
	334	2.28	0.27	0.00	nd	-	2.60 C1	0.04	4	
	85	2.14	0.19	0.02	nd	-	2.62 C1	0.04	1,2,3,4,5	
	117	7.03	0.23	0.02	nd	-	2.68 C1	0.02	1,2,5	
	55	1.24	0.10	0.01	nd	-	2.70 C1	0.01	0	
	129	2.57	0.12	0.00	nd	-	2.73 C1	0.05	 	
	18	1.81	0.31	0.00	0.02	0.00	2.76 C1	0.05	 	
	89	2.47	0.23	0.01	0.01	0.00	2.77 C1	0.02	 	
	13	3.52	0.13	0.02	nd	-	2.80 C1	0.03	 	
	74	2.27	0.25	0.02	0.01	0.00	2.83 C2	0.06	 	
	93	1.05	0.09	0.01	nd	-	2.84 C2	0.06	 	
	141	8.28	0.21	0.00	nd	-	2.85 C2	0.04	 	
	20	1.39	0.08	0.00	nd	-	2.86 C2	0.02	1,2,4,5	
	44	2.73	0.33	0.03	nd	-	2.88 C2	0.08	1,3,4	
	368	1.85	0.42	0.02	0.03	0.00	2.89 C2	0.06	0	
	347	1.27	0.07	0.01	nd	-	2.90 C2	1.91	 	
	63	7.40	0.22	0.02	0.01	0.00	2.99 C2	0.01	 	
	150	3.77	0.38	0.03	0.01	0.00	2.99 C2	0.01	 	
	391	1.18	0.10	0.00	nd	-	3.01 C2	0.05	1,2,3,4,5	
	131	0.13	0.16	0.01	0.00	0.00	3.02 C2	0.02	1,2,4,5	
	67	1.38	0.28	0.04	0.02	0.00	3.03 C2	0.09	 	
	75	2.29	0.49	0.04	0.02	0.00	3.04 C2	0.08	0	
	127	1.32	0.37	0.02	0.03	0.00	3.13 C2	0.09	1,2,4	
	88	0.42	0.14	0.01	nd	-	3.19 C2	0.07	1,2,5	
	62	0.19	0.05	0.00	nd	-	3.25 C2	0.00	 	
	300	0.94	0.12	0.01	nd	-	3.31 C2	0.04	 	
	283	1.52	0.28	0.04	nd	-	3.33 C2	0.10	 	
	65	3.12	0.27	0.01	0.01	0.00	3.36 C2	0.11	 	
	8	2.64	0.61	0.07	0.03	0.00	3.38 C2	0.03	1,2,4,5	
	257	0.78	0.08	0.01	nd	-	3.39 C2	0.03	 	
	7	1.65	0.22	0.01	0.02	0.00	3.41 C2	0.08	 	
	128	0.63	0.05	0.00	0.07	0.00	3.42 C2	0.34	1,2,5	
	207	0.64	0.09	0.00	nd	-	3.47 C2	0.03	 	
	11	0.98	0.21	0.01	nd	-	3.58 C2	0.01	1,2,4,5	
	222	0.77	0.05	0.01	nd	-	3.67 C2	0.08	 	
	98	0.29	0.12	0.00	0.05	0.00	4.08 C3	0.05	1,2,3,4,5	
	322	0.69	0.03	0.00	nd	-	4.24 C3	0.09	 	
	276	0.39	0.04	0.00	nd	-	4.25 C3	0.01	 	
	241	1.28	0.05	0.01	nd	-	4.33 C3	0.01	 	
	266	0.22	0.08	0.01	nd	-	4.42 C3	0.04	 	
	23	2.18	0.23	0.02	nd	-	4.61 C3	0.16	 	
	115	2.45	0.18	0.02	nd	-	4.63 C3	0.24	 	
	281	2.44	0.18	0.02	nd	-	4.88 C3	0.10	 	
	76	1.95	0.20	0.01	nd	-	4.89 C3	0.16	 	
	91	0.99	0.10	0.01	0.02	0.00	5.06 C3	0.36	 	
	252	1.45	0.08	0.00	nd	-	5.29 C3	0.04	 	
	10	2.09	0.31	0.02	nd	-	5.46 C3	0.35	1,2,4,5	
	231	1.34	0.16	0.01	nd	-	5.82 C3	0.47	 	
	*173	1.31	0.05	0.00	0.04	0.00	6.12 C3	0.79	1,2,3,4,5	
	311	1.76	0.25	0.02	0.17	0.01	7.20 C4	0.33	1,2,4,5	
	154	0.85	0.18	0.01	0.04	0.00	7.33 C4	0.83	1	
	*95	2.99	0.11	0.01	1.61	0.09	7.50 C4	0.28	1,2,3,4,5	
	*132	0.45	0.10	0.01	0.04	0.00	7.56 C4	0.14	1,2,3,4,5	
	182	2.55	0.11	0.00	0.05	0.00	9.04 C5	0.27	1,4,5	

AO3-CS	15	0.37	nd	-	nd	-	nd Cnd	-	1,2,4,5	
	36	0.23	0.14	0.02	nd	-	nd Cnd	-	 	
	143	0.74	0.21	0.01	0.01	0.00	1.87 C1	0.17	 	
	175	0.60	0.09	0.00	nd	-	2.52 C1	0.08	 	
	19	0.24	0.18	0.01	nd	-	2.73 C1	0.03	1,2,4,5	
	219	0.33	0.14	0.01	0.81	0.08	2.77 C1	0.00	2,4,5	
	160	1.62	0.15	0.01	0.03	0.00	2.84 C1	0.08	 	
	302	2.04	0.12	0.00	nd	-	2.85 C1	0.03	1	
	146	2.09	0.29	0.01	0.02	0.00	2.90 C1	0.15	 	
	38	1.65	0.20	0.01	0.03	0.00	2.95 C1	0.02	 	
	158	0.07	0.06	0.00	nd	-	3.02 C1	0.04	 	
	353	0.37	0.11	0.01	nd	-	3.12 C1	0.07	0	
	141	1.11	0.24	0.01	nd	-	3.14 C1	0.18	 	
	66	0.46	0.17	0.01	0.01	0.00	3.16 C1	0.08	 	
	124	0.29	0.14	0.01	nd	-	3.21 C1	0.18	1,2,4	
	150	0.98	0.23	0.01	0.04	0.00	3.27 C1	0.09	 	
	218	0.45	0.17	0.01	0.05	0.00	3.31 C1	0.05	1,2,5	
	406	0.12	0.07	0.00	nd	-	3.31 C1	0.09	1,2,4,5	
	117	1.60	0.25	0.02	0.01	0.00	3.33 C1	0.17	 	
	57	0.89	0.33	0.01	0.03	0.00	3.36 C1	0.06	1,3,4,5	
	90	0.93	0.26	0.02	0.02	0.00	3.36 C1	0.11	 	
	23	0.76	0.28	0.02	0.02	0.00	3.41 C1	0.08	 	
	99	0.51	0.32	0.04	0.02	0.00	3.42 C1	0.27	 	
	380	1.20	0.16	0.01	nd	-	3.43 C1	0.04	 	
	276	0.09	0.03	0.00	0.04	0.00	3.46 C1	0.11	1,2,4?,5	
	128	0.58	0.46	0.03	0.03	0.00	3.47 C1	0.10	 	
	313	0.71	0.32	0.02	0.03	0.01	3.48 C1	0.09	1,2,3,4,5	
	118	0.30	0.12	0.01	nd	-	3.56 C1	0.27	 	
	213	1.34	0.41	0.01	0.02	0.00	3.58 C1	0.05	 	
	129	1.41	0.50	0.03	0.03	0.00	3.59 C1	0.10	 	
	110	9.39	0.66	0.01	0.03	0.00	3.63 C1	0.09	2,5	
	27	1.77	0.43	0.03	0.03	0.00	3.63 C1	0.04	 	
	425	0.12	0.03	0.00	nd	-	3.66 C1	0.06	1,2,5	
	277	0.62	0.25	0.01	nd	-	3.69 C1	0.15	1,2,4,5	
	193	0.25	0.21	0.01	nd	-	3.76 C1	0.16	1,2,4,5	
	78	0.56	0.08	0.00	nd	-	3.76 C1	0.17	1,2,4,5	
	230	0.69	0.09	0.02	nd	-	3.88 C1	0.52	 	
	131	0.17	0.14	0.01	0.01	0.00	3.89 C1	0.12	 	
	284	1.98	0.27	0.01	0.03	0.00	3.90 C1	0.15	1,3,4	
	274	0.13	0.10	0.01	0.01	0.00	3.92 C1	0.10	 	
	102	0.51	0.10	0.01	0.02	0.00	3.93 C1	0.04	1,2,5	
	6	0.76	0.35	0.05	0.02	0.00	3.96 C1	0.23	 	
	402	0.25	0.11	0.01	nd	-	3.97 C1	0.06	1,2,3,4,5	
	40	1.01	0.43	0.10	0.04	0.00	4.00 C1	0.34	0	
	159	2.16	0.26	0.04	nd	-	4.04 C1	0.43	 	
	106	0.52	0.09	0.01	nd	-	4.07 C1	0.16	2,5	
	89	0.71	0.27	0.07	nd	-	4.09 C1	0.19	 	
	247	0.48	0.04	0.00	nd	-	4.12 C1	0.14	 	
	46	0.23	0.51	0.05	0.05	0.01	4.14 C1	0.11	1,4,5	
	270	4.69	0.17	0.03	0.01	0.00	4.15 C1	0.15	 	
	419	1.29	0.25	0.01	0.01	0.00	4.30 C1	0.03	 	
	290	0.41	0.13	0.01	nd	-	4.32 C1	0.10	 	
	45	1.91	0.33	0.00	0.02	0.00	4.35 C1	0.33	1,2	
	384	0.50	0.34	0.02	0.03	0.00	4.45 C1	0.10	0	
	115	0.44	0.39	0.02	0.03	0.00	4.48 C1	0.12	1	
	50	0.12	0.15	0.01	0.03	0.00	4.52 C1	0.46	1,2,4,5	
	132	1.34	0.31	0.05	0.03	0.00	4.52 C1	0.73	 	
	149	0.31	0.13	0.02	0.01	0.00	4.54 C1	0.17	1,2,4,5	
	281	1.06	0.10	0.01	nd	-	4.56 C1	0.03	 	
	306	0.61	0.42	0.08	nd	-	4.69 C1	0.23	 	
	330	0.78	0.16	0.02	nd	-	4.73 C1	0.32	 	
	348	0.12	0.05	0.00	nd	-	4.78 C1	0.22	 	
	401	0.17	0.05	0.02	nd	-	4.79 C1	0.07	 	
	153	1.38	0.07	0.02	nd	-	4.90 C1	0.05	 	
	127	0.42	0.32	0.04	0.02	0.00	4.91 C1	0.18	 	
	372	1.21	0.16	0.02	nd	-	4.92 C1	0.04	 	
	420	1.13	0.10	0.00	nd	-	4.98 C1	0.11	 	
	399	0.84	0.09	0.00	nd	-	5.03 C1	0.04	 	
	25	1.12	0.19	0.02	0.03	0.01	5.11 C1	0.21	 	
	60	0.67	0.47	0.06	0.03	0.01	5.14 C1	0.60	 	
	221	1.19	0.19	0.01	0.04	0.00	5.16 C1	0.02	 	
	69	0.99	0.43	0.03	0.02	0.00	5.18 C1	0.01	1,2,4,5	
	325	0.46	0.09	0.01	nd	-	5.21 C1	0.03	 	
	93	0.64	0.10	0.01	0.01	0.00	5.26 C1	0.08	 	
	388	nd	0.04	0.00	nd	-	5.28 C1	0.16	1	
	394	0.53	0.17	0.01	nd	-	5.30 C1	0.01	1,5	
	392	0.43	0.10	0.00	0.01	0.00	5.37 C1	0.01	 	
	63	0.59	0.48	0.02	0.04	0.00	5.38 C1	0.26	 	
	162	0.34	0.13	0.01	nd	-	5.40 C1	0.12	 	
	83	0.74	0.20	0.01	nd	-	5.50 C1	0.14	 	
	269	0.57	0.22	0.01	0.13	0.01	5.62 C2	0.16	1,2,3,4,5	
	386	0.87	0.13	0.01	0.01	0.00	5.68 C2	0.16	 	
	262	1.22	0.56	0.06	0.06	0.01	5.71 C2	0.11	1,2,4,5	
	400	0.46	0.13	0.01	nd	-	5.84 C2	0.22	 	
	201	0.59	0.26	0.03	0.07	0.01	5.89 C2	0.32	1,2,3,4,5	
	191	0.40	0.33	0.03	0.02	0.00	5.91 C2	0.10	1,4,5	
	329	1.18	0.30	0.03	0.01	0.00	6.01 C2	0.32	 	
	22	1.75	0.39	0.07	nd	-	6.07 C2	0.66	 	
	12	0.83	0.15	0.01	0.04	0.00	6.15 C2	0.11	1,2,4,5	
	198	1.33	0.61	0.04	0.05	0.01	6.18 C2	0.19	1,2,3,4,5	
	355	0.31	0.11	0.01	nd	-	6.21 C2	0.25	1,4	
	318	0.43	0.18	0.00	0.02	0.00	6.49 C2	0.11	1,4,5	
	171	0.60	0.33	0.02	0.11	0.00	6.66 C2	0.24	1,2,3,4,5	
	272	0.79	0.18	0.02	0.01	0.01	6.95 C2	0.06	 	
	345	0.18	0.13	0.03	nd	-	7.53 C2	0.34	1,2	
	87	0.29	0.09	0.01	nd	-	7.58 C2	0.04	 	
	*84	0.44	0.28	0.05	0.02	0.00	7.72 C2	0.67	1,2,4	
	125	0.97	0.10	0.01	0.21	0.03	7.75 C2	0.02	1,2,3,4,5	
	*145	5.05	0.23	0.04	0.11	0.02	8.01 C2	0.71	1,2,3,4,5	
	310	0.50	0.21	0.02	0.06	0.00	8.14 C2	0.35	1,4,5	
	166	1.04	0.05	0.00	0.02	0.00	8.23 C2	0.06	 	
	383	nm	0.06	0.00	nd	-	8.28 C2	0.22	 	
	86	1.38	0.38	0.04	0.05	0.00	8.36 C2	0.52	1,2,3,4,5	
	*147	2.50	0.12	0.01	0.07	0.01	8.39 C2	0.29	1,2,3,4,5	
	52	1.30	0.28	0.01	0.03	0.00	8.44 C2	0.58	1,2,3,4,5	
	114	0.86	0.55	0.05	0.06	0.00	10.34 C2	1.20	1,2,4,5	
	*144	0.50	0.17	0.00	0.02	0.00	12.86 C3	0.13	1,2,4,5	
	189	0.36	0.08	0.00	nd	-	13.33 C3	0.63	1,2,3,4,5	
	210	1.34	0.13	0.01	nd	-	13.58 C3	0.29	1,2,3,4,5	
	356	0.06	0.03	0.01	nd	-	13.73 C3	1.43	1,4	
	126	0.24	0.29	0.02	0.03	0.00	14.26 C3	0.60	 	
	389	0.27	0.06	0.00	0.02	0.00	14.69 C3	1.92	1,2,4,5	
	316	0.59	0.33	0.01	0.11	0.02	15.38 C3	0.65	1,2,4,5	
	*53	0.31	0.14	0.00	0.09	0.01	19.05 C3	0.84	2,4?,5	
	*58	0.67	0.19	0.01	0.04	0.01	22.52 C4	1.39	1,2,4	
	121	0.71	0.14	0.01	nd	-	23.47 C4	1.42	1,2,3,4,5	
	250	1.83	0.10	0.00	nd	-	25.94 C4	0.57	1,2,4,5	
	*336	0.49	nd	-	nd	-	30.86 C4	0.81	1,2,4,5	
	424	1.04	0.07	0.01	0.02	0.00	31.58 C4	1.68	1,2,4,5	
	*180	1.57	0.01	0.00	0.01	0.00	75.85 C5	3.48	1,2,3,4,5	
