# Supplementary material for: A new synthetic biology approach allows transfer of an entire metabolic pathway from a medicinal plant to a biomass crop
Source: eLife. 2016 Jun 14;5:e13664. doi: 10.7554/eLife.13664 (PMC4907697; doi:10.7554/eLife.13664)
Supplement: Related texts

# Article commentary: Decision letter

**DOI:** 10.7554/eLife.13664.019

**Affiliations:** University of British Columbia, Canada

In the interests of transparency, eLife includes the editorial decision letter and accompanying author responses. A lightly edited version of the letter sent to the authors after peer review is shown, indicating the most substantive concerns; minor comments are not usually included.

Thank you for submitting your work entitled "A new synthetic biology approach allows transfer of an entire metabolic pathway from a medicinal plant to a biomass crop" for consideration by *eLife*. Your article has been reviewed by two peer reviewers, and the evaluation has been overseen by a Reviewing Editor and Ian Baldwin as the Senior Editor.

Both reviewers liked the topic and scope of the paper and found it to be of sufficient broad interest and importance for the audience of *eLife*. The Reviewing Editor agrees with this positive general assessment. However, both reviewers also expressed a number of concerns, which need to be addressed with major revisions.

The reviewers have discussed the reviews with one another and the Reviewing Editor has drafted this recommendation, which has also been seen and approved by both reviewers, to help you prepare a revised submission.

Summary of the work

There are many plant natural products of value in medicine, but few are as valuable as the anti-malarial sesquiterpene artemisinin, which is limited in supply due to production only in specialized anatomical structures, the glandular trichomes, of the Chinese medicinal plant *Artemisia annua*. This situation has stimulated many research projects to try to increase artemisinin supply in plant and microbial systems, and much has been learned about metabolic engineering and the regulation of isoprenoid biosynthesis along the way.

The present manuscript reports on a new approach to increasing artemisinin formation by transferring most of the pathway for artemisinic acid into the chloroplast genome of tobacco. By first developing tobacco lines that express the core pathway genes/enzymes and then selecting lines from a population containing different combinations and expression levels of other (accessory) pathway genes/ enzymes, the authors achieved a remarkable increase in the yield of artemisinic acid, an advanced intermediate that can be chemically converted to artemisinin.

Previous publications reported nuclear transformation of artemisin biosynthetic genes. The present manuscript is apparently the first report of a systematic and comprehensive study on the possibility to produce artemisinin precursors through chloroplast transformation. The work appears to be novel and of substantial merit. It provides interesting new basic information on pathway regulation and similar approaches should be applicable to a range of other metabolic engineering problems. The value of this work has not been oversold, and for most parts of the manuscript, the research has been carried out at a high level of quality.

The reviewers have a number of constructive suggestions to improve the presentation of the paper, requests for more information, and corrections.

The combined set of comments from the two reviewers that require major revisions are following below.

Essential major revisions:

1) Based on the data presented, it is difficult to claim that the ratio of CYP and CPR is a critical factor determining the yield of artemisinic acid (subsection “Expression of the core pathway for artemisinic acid synthesis from the plastid genome”, last paragraph). Northern blot data in Figure 4(D-G) are ambiguous and are not labeled for the specific transcript (FPS, ADS, CYP, CPR). There appear to be multiple signals likely due to non-specific probe hybridization. As for the qRT-PCR used for COSTRL analysis, qRT-PCR should be used to compare the relative CYP and CPR transcript level. It is also important to note that, transcript levels do not necessarily reflect levels of the corresponding protein or enzyme activity. The claims in the aforementioned paragraph need to be stated with much caution.

2) DBR (double-bond reductase) is a required enzyme for artemisinin biosynthesis, and it is not correct to refer to DBR as an accessory enzyme. Since DBR diverts the flux from artemisinin acid to dihydroartemisinic acid, the authors should measure dihydroartemisinic acid for the lines expressing DBR shown in Figure 5. Curiously, dihydroartemisinic alcohol was measured (Figure 5—source data 1). To produce artemisinin from artemisinic acid, an additional chemical process, diastereoselective hydrogenation of artemisinic acid, is necessary, and therefore dihydroartemisinic acid is considered as an advanced form of artemisinin precursor, compared to artemisinic acid. The readers will want to know how much dihydroartemisinic acid is synthesized from the lines transformed with DBR. If dihydroartemisinic acid could not be detected, it has to be explicitly stated and discussed. Along the same line, it is currently believed that artemisinin can be synthesized non-enzymatically from dihydroartemisinic acid. Thus, the last paragraph in subsection "Identification of limiting steps in artemisinic acid biosynthesis" cannot be stated without including dihydroartemisinic acid data (artemisinic acid could be non-enzymatically converted to arteannuin B but not to arteminin). If the authors transform tobacco with DBR, dihydroartemisinic acid needs to be measured and quantified.

3) It would be important and interesting to know if the production of this novel sesquiterpene in tobacco caused any trade-offs with growth or development. The figures give us what are said to be representative pictures of the wild-type and various transgenic lines that show no obvious yield penalty for transgenic sesquiterpene production. But, it would be useful if growth were quantified more rigorously (height, leaf area, total biomass) to obtain averages from multiple individuals. Was this done? This could be crucial for the economic value of such transgenic lines.

4) In previous efforts of metabolic engineering of terpenoids in tobacco, a large fraction of glycosylated terpene metabolites has sometimes been detected. Since the artemisinin pathway has hydroxylated and carboxylated intermediates, which could be converted to glycosylated products, the authors should look for such compounds, which could potentially represent a large percentage of pathway flux. This might require a more polar extraction solvent, methanol, followed by cleavage with acid or β-glucosidase). Knowledge of the occurrence of glycosylated artemisinin pathway derivatives would give a more complete picture of the metabolic changes resulting from the transgenes and might reveal a large pool of intermediates that could be eventually recovered for artemisinin production by blocking such conjugation.

5) Much of the Discussion is not directly related to the core of the results presented in this paper and was generally found to be one of the weakest parts of the paper. The Discussion should be rewritten and focused. Some specific suggestions for improving of the discussion:

i) The authors' new results should be discussed in the context of other successful transplastomics work as it relates to plant secondary metabolism (e.g., 5 mg/g astaxanthin reported by Hasunuma et al., 2008, Plant J. 55, 857).

ii) It is now well established in the field of metabolic engineering that balanced enzyme activities are more important than levels of overexpression. See for example work on microbial metabolic engineering by the Keasling group. The approach described in the present paper is the first such attempt in plant metabolic engineering, and therefore authors could discuss the importance of balancing the activities of metabolic enzymes by using COSTRL.

iii) If the authors could detect dihydroartemisinic acid, the absence of artemisinin would in fact be an interesting result, because chloroplasts should have sufficient oxygen and reactive oxygen. This could be further discussed in comparison to other published work on artemisinin bioengineering. According to the Methods section, dihydroartemisinic acid was used as an authentic standard to confirm dihydroartemisinic acid in plants. This raises the question as to why dihydroartemisinic acid has not been discussed much, while DBR or artemisinin formation by non-enzymatic reaction are discussed.

iv) Some plant P450s localize to plastids but their redox partner in the chloroplast remains poorly understood. Ferredoxin and ferredoxin reductase may provide reducing equivalents to plastid localized P450s. Apparently, AMO operates well in the chloroplast based on the product formation. With fovus on the engineering objectives, it may not matter how a normally ER-localized P450 can efficiently operate in chloroplasts. However, this question needs to be discussed.

We would not require the authors to provide any of the additional data asked for in the points below, but these would be very useful in supporting some of the interpretations and speculations in the paper. And at the minimum are additional points for discussion:

6) Did the authors check if the pathway operates only in chloroplasts or also in non-photosynthetic plastids in leaves, stems or roots? It might be very useful to know where artemisinic acid accumulates. If accumulation is linked with photosynthesis that might help interpret the results of the CYP/CPR expression data since the frequency and consequences of CPR activation with oxygen might be very different between photosynthetic and non-photosynthetic cells.

7) A number of metabolic results in this manuscript are explained by reference to transcript levels of the transgenes. Did the authors ever perform any enzyme assays to determine the corresponding activities? Changes in the activity of the CYP enzyme might be relevant to explaining the effects of different CYP/CPR transcript ratios.

8) The native artemisinin pathway is localized in glandular trichomes. Since tobacco also has glandular trichomes, albeit of a very different type, it might be worthwhile for the authors to look for the artemisinic acid in the glandular trichome secretion of their transgenic lines. Did they try this? Also, the glandular trichomes of tobacco may well resemble the cellular environment of artemisinin biosynthesis in the Artemisia, and artemisinin might be produced in trichomes by non-enzymatic conversion from dihydroartemisinic acid. Has this been tested? Was there any attempt to try and localize artemisinin intermediate accumulation at the tissue and cell level?

# Reply: Author response

**DOI:** 10.7554/eLife.13664.020

1) Based on the data presented, it is difficult to claim that the ratio of CYP and CPR is a critical factor determining the yield of artemisinic acid (subsection “Expression of the core pathway for artemisinic acid synthesis from the plastid genome”, last paragraph). Northern blot data in Figure 4(D-G) are ambiguous and are not labeled for the specific transcript (FPS, ADS, CYP, CPR). There appear to be multiple signals likely due to non-specific probe hybridization. As for the qRT-PCR used for COSTRL analysis, qRT-PCR should be used to compare the relative CYP and CPR transcript level. It is also important to note that, transcript levels do not necessarily reflect levels of the corresponding protein or enzyme activity. The claims in the aforementioned paragraph need to be stated with much caution.

The presence of multiple bands in the northern blot experiments is not due to non-specific probe hybridization. Instead, the larger bands represent unprocessed polycistronic precursor transcripts and read-through transcripts (which are common in plastids; e.g., Elghabi et al., 2011, Lu et al., 2013). This is now stated in the figure legend. To provide additional evidence for the different CYP:CPR ratios, we have performed the suggested qRT-PCR assays for all tissues and developmental stages. The data fully confirm the northern blot data and are displayed in Figure 4—figure supplement 1 of the revised manuscript. As suggested, we have also added a note of caution to the discussion of these data, stating that determination of the CYP and CPR protein accumulation levels (and enzyme activities) would be required to precisely assess these ratios and ultimately confirm their impact on metabolite conversion in the pathway.

2) DBR (double-bond reductase) is a required enzyme for artemisinin biosynthesis, and it is not correct to refer to DBR as an accessory enzyme. Since DBR diverts the flux from artemisinin acid to dihydroartemisinic acid, the authors should measure dihydroartemisinic acid for the lines expressing DBR shown in Figure 5. Curiously, dihydroartemisinic alcohol was measured (Figure 5—source data 1). To produce artemisinin from artemisinic acid, an additional chemical process, diastereoselective hydrogenation of artemisinic acid, is necessary, and therefore dihydroartemisinic acid is considered as an advanced form of artemisinin precursor, compared to artemisinic acid. The readers will want to know how much dihydroartemisinic acid is synthesized from the lines transformed with DBR. If dihydroartemisinic acid could not be detected, it has to be explicitly stated and discussed. Along the same line, it is currently believed that artemisinin can be synthesized non-enzymatically from dihydroartemisinic acid. Thus, the last paragraph in subsection "Identification of limiting steps in artemisinic acid biosynthesis" cannot be stated without including dihydroartemisinic acid data (artemisinic acid could be non-enzymatically converted to arteannuin B but not to arteminin). If the authors transform tobacco with DBR, dihydroartemisinic acid needs to be measured and quantified.

As suggested, we have measured dihydroartemisinic acid in all plant lines and samples. Interestingly, the compound was detectable only in two of our best-performing COSTREL lines. The data are now included in the revised Figure 7 and Figure 5—source data 1 and Figure 5—figure supplement 1, and discussed in the text (subsection “Identification of limiting steps in artemisinic acid biosynthesis”). We also agree with the point made by the reviewer(s) that DBR2 is not an accessory enzyme sensu stricto, and we have rephrased the corresponding sentences in the manuscript.

3) It would be important and interesting to know if the production of this novel sesquiterpene in tobacco caused any trade-offs with growth or development. The figures give us what are said to be representative pictures of the wild-type and various transgenic lines that show no obvious yield penalty for transgenic sesquiterpene production. But, it would be useful if growth were quantified more rigorously (height, leaf area, total biomass) to obtain averages from multiple individuals. Was this done? This could be crucial for the economic value of such transgenic lines.

As suggested, we have performed comparative growth experiments with a large number of plants and quantified key growth parameters (plant height, total leaf biomass). The data confirmed that there are no significant differences between transplastomic line *Nt*-AO3-1 and the best-performing COSTREL line *Nt*-AO3-CS180 (and revealed only a small reduction in total leaf biomass in all *Nt*-AO3 plants compared to the wild type). These data are presented in Figure 5—figure supplement 2 and discussed in the text.

4) In previous efforts of metabolic engineering of terpenoids in tobacco, a large fraction of glycosylated terpene metabolites has sometimes been detected. Since the artemisinin pathway has hydroxylated and carboxylated intermediates, which could be converted to glycosylated products, the authors should look for such compounds, which could potentially represent a large percentage of pathway flux. This might require a more polar extraction solvent, methanol, followed by cleavage with acid or β-glucosidase). Knowledge of the occurrence of glycosylated artemisinin pathway derivatives would give a more complete picture of the metabolic changes resulting from the transgenes and might reveal a large pool of intermediates that could be eventually recovered for artemisinin production by blocking such conjugation.

The reviewers point out correctly that terpenoids, including artemisinic compounds, can be partially glycosylated. Our isolation protocols capture both the glycosylated and the non-glycosylated intermediates in the artemisinin pathway. To obtain realistic values for the flux through the pathway, we determined the sum of free and conjugated compounds (by GC-MS analysis of the soluble metabolite fraction after saponification). This is now explicitly stated in the legends to Figures 3 and 4. The relevant procedures are described in the Materials and methods section.

5) Much of the Discussion is not directly related to the core of the results presented in this paper and was generally found to be one of the weakest parts of the paper. The Discussion should be rewritten and focused. Some specific suggestions for improving of the discussion:

We have extensively revised the Discussion section of the manuscript. The four specific suggestions were incorporated as follows:

i) The authors' new results should be discussed in the context of other successful transplastomics work as it relates to plant secondary metabolism (e.g., 5 mg/ g astaxanthin reported by Hasunuma et al., 2008, Plant J. 55, 857).

We now provide a summary of previous metabolic engineering efforts using transplastomic technologies. We discuss examples where endogenous metabolic pathways were enhanced and examples where novel metabolites were produced (including the ketocarotenoid work by Hasunuma et al.; Discussion, fourth paragraph).

ii) It is now well established in the field of metabolic engineering that balanced enzyme activities are more important than levels of overexpression. See for example work on microbial metabolic engineering by the Keasling group. The approach described in the present paper is the first such attempt in plant metabolic engineering, and therefore authors could discuss the importance of balancing the activities of metabolic enzymes by using COSTRL.

We agree that establishing the optimum balance of enzyme activities is often more critical than absolute (over)expression levels. As suggested, this is now discussed in the context of large-scale metabolic engineering efforts in microbial systems and the significant advantages that our COSTREL strategy provides with achieving that optimum balance (Discussion section).

iii) If the authors could detect dihydroartemisinic acid, the absence of artemisinin would in fact be an interesting result, because chloroplasts should have sufficient oxygen and reactive oxygen. This could be further discussed in comparison to other published work on artemisinin bioengineering. According to the Methods section, dihydroartemisinic acid was used as an authentic standard to confirm dihydroartemisinic acid in plants. This raises the question as to why dihydroartemisinic acid has not been discussed much, while DBR or artemisinin formation by non-enzymatic reaction are discussed.

See point 2. The detection of DHAA is now described and the data are shown in Figures 7 and Figure 5—source data 1 and Figure 5—figure supplement 1. We discuss the low accumulation of DHAA and the absence of artemisinin (and possible reasons) in the subsection “Identification of limiting steps in artemisinic acid biosynthesis”.

iv) Some plant P450s localize to plastids but their redox partner in the chloroplast remains poorly understood. Ferredoxin and ferredoxin reductase may provide reducing equivalents to plastid localized P450s. Apparently, AMO operates well in the chloroplast based on the product formation. With fovus on the engineering objectives, it may not matter how a normally ER-localized P450 can efficiently operate in chloroplasts. However, this question needs to be discussed.

As suggested, we also discuss how P450 enzymes (that normally reside in the ER membrane) can function in chloroplasts. In particular, we discuss recent evidence that these enzymes, when anchored to the thylakoid membrane, can be reduced directly by electrons from the photosynthetic electron transfer chain, presumably with reduced ferredoxin acting as electron donor (Gnanasekaran et al., 2016; Discussion section, fourth paragraph).

We would not require the authors to provide any of the additional data asked for in the points below, but these would be very useful in supporting some of the interpretations and speculations in the paper. And at the minimum are additional points for discussion:

6) Did the authors check if the pathway operates only in chloroplasts or also in non-photosynthetic plastids in leaves, stems or roots? It might be very useful to know where artemisinic acid accumulates. If accumulation is linked with photosynthesis that might help interpret the results of the CYP/CPR expression data since the frequency and consequences of CPR activation with oxygen might be very different between photosynthetic and non-photosynthetic cells.

We have not measured metabolite accumulation in non-green tissues, because expression of chloroplast transgenes is known to be very low in non-green plastid types. Previous work has shown that significant levels of transgene expression in non-green plastids can only be achieved by designing specific (chimeric) expression signals that are active in non-photosynthetic tissues (Zhang et al., 2012; Caroca et al., 2013). This is now discussed (and referenced) in the revised manuscript.

7) A number of metabolic results in this manuscript are explained by reference to transcript levels of the transgenes. Did the authors ever perform any enzyme assays to determine the corresponding activities? Changes in the activity of the CYP enzyme might be relevant to explaining the effects of different CYP/CPR transcript ratios.

We currently do not have antibodies or suitable enzyme activity assays to confirm the significance of the different CYP/CPR ratios at the post-transcriptional level. This and the possibility of post-transcriptional regulation influencing the regulation of CYP activity are now mentioned at the end of the subsection “Expression of the core pathway for artemisinic acid synthesis from the plastid 98 genome”.

8) The native artemisinin pathway is localized in glandular trichomes. Since tobacco also has glandular trichomes, albeit of a very different type, it might be worthwhile for the authors to look for the artemisinic acid in the glandular trichome secretion of their transgenic lines. Did they try this? Also, the glandular trichomes of tobacco may well resemble the cellular environment of artemisinin biosynthesis in the Artemisia, and artemisinin might be produced in trichomes by non-enzymatic conversion from dihydroartemisinic acid. Has this been tested? Was there any attempt to try and localize artemisinin intermediate accumulation at the tissue and cell level?

See point 5. Although tobacco leaves also possess glandular trichomes, the trichomes in our COSTREL plants are unlikely to accumulate large amounts of artemisinic acid. This is because transgene expression from the plastid genome is generally very low in non-photosynthetic tissues and cell types. It can be significantly enhanced by designing specific (chimeric) expression signals that confer high transgene activity in non-green tissues (Zhang et al., 2012, Caroca et al., 2013), but the expression signals used to drive our synthetic artemisinic acid operons (Figure 2) were chosen to maximize expression in chloroplasts and, therefore, are not suitable to trigger efficient gene expression in non-photosynthetic plastids. This is explained in the third paragraph of the Discussion section.
